# Transcriptional networks orchestrating red and pink testa color in peanut

**DOI:** 10.1186/s12870-023-04041-0

**Published:** 2023-01-19

**Authors:** Naveed Ahmad, Kun Zhang, Jing Ma, Mei Yuan, Shuzhen Zhao, Mingqing Wang, Li Deng, Li Ren, Sunil S. Gangurde, Jiaowen Pan, Changle Ma, Changsheng Li, Baozhu Guo, Xingjun Wang, Aiqin Li, Chuanzhi Zhao

**Affiliations:** 1 Institute of crop germplasm resources (Institute of Biotechnology), Shandong Academy of Agricultural Sciences; Shandong Provincial Key Laboratory of Crop Genetic Improvement, Ecology and Physiology, Jinan, 250100 People’s Republic of China; 2 Joint Center for Single Cell Biology, School of Agriculture and Biology, Shanghai Jiao Tong University, Shanghai, 200240 China; 3 College of Agricultural Science and Technology, Shandong Agriculture and Engineering University, Jinan, 250100 People’s Republic of China; 4 College of Life Sciences, Shandong Normal University, Jinan, 250014 People’s Republic of China; 5 Shandong Peanut Research Institute, Qingdao, 266199 Shandong People’s Republic of China; 6 Kaifeng Academy of Agriculture and Forestry, Kaifeng, 475008 People’s Republic of China; 7 Crop Protection and Management Research Unit, USDA-ARS, Tifton, GA 31793 USA; 8 Department of Plant Pathology, University of Georgia, Tifton, GA 31793 USA

**Keywords:** Peanut, Testa color, Anthocyanin biosynthesis, flavonoids, Bulk RNA-seq (BR-seq), Transcription factors

## Abstract

**Background:**

Testa color is an important trait of peanut (*Arachis hypogaea* L.) which is closely related with the nutritional and commercial value. Pink and red are main color of peanut testa. However, the genetic mechanism of testa color regulation in peanut is not fully understood. To elucidate a clear picture of peanut testa regulatory model, samples of pink cultivar (Y9102), red cultivar (ZH12), and two RNA pools (bulk red and bulk pink) constructed from F_4_ lines of Y9102 x ZH12 were compared through a bulk RNA-seq approach.

**Results:**

A total of 2992 differential expressed genes (DEGs) were identified among which 317 and 1334 were up-regulated and 225 and 1116 were down-regulated in the bulk red-vs-bulk pink RNA pools and Y9102-vs-ZH12, respectively. KEGG analysis indicates that these genes were divided into significantly enriched metabolic pathways including phenylpropanoid, flavonoid/anthocyanin, isoflavonoid and lignin biosynthetic pathways. Notably, the expression of the anthocyanin upstream regulatory genes *PAL*, *CHS*, and *CHI* was upregulated in pink and red testa peanuts, indicating that their regulation may occur before to the advent of testa pigmentation. However, the differential expression of down-stream regulatory genes including *F3H*, *DFR*, and *ANS* revealed that deepening of testa color not only depends on their gene expression bias, but also linked with *FLS* inhibition. In addition, the down-regulation of *HCT*, *IFS*, *HID*, 7-*IOMT*, and *I2’H* genes provided an alternative mechanism for promoting anthocyanin accumulation via perturbation of lignin and isoflavone pathways. Furthermore, the co-expression module of MYB, bHLH, and WRKY transcription factors also suggested a fascinating transcriptional activation complex, where MYB-bHLH could utilize WRKY as a co-option during the testa color regulation by augmenting anthocyanin biosynthesis in peanut.

**Conclusions:**

These findings reveal candidate functional genes and potential strategies for the manipulation of anthocyanin biosynthesis to improve peanut varieties with desirable testa color.

**Supplementary Information:**

The online version contains supplementary material available at 10.1186/s12870-023-04041-0.

## Background

Anthocyanin is an important color pigment, which is widely distributed across the plant kingdom. The accumulation of anthocyanin pigments including pelargonidin, cyanidin, dalphinidin, petudinin, and malvidin largely contribute to the pink, red, blue, and purple colors found in plant tissues such as flowers, leaves, fruits, and roots [1]. In addition to their important roles as pollinator and insect attractant, anthocyanins can also assist plants against abiotic stresses such as cold, drought stress, and ultraviolet irradiation as well as pathogenic microbes [2, 3]. Anthocyanins and its derivatives are usually believed to possess health-promoting properties through high antioxidant activities, allowing plants to combat ROS-induced risks [4]. Regular consumption of anthocyanins has been shown to lessen the chance of developing atherosclerosis and cardiovascular diseases by suppressing the oxidation of low-density lipids. Previously, many studies have demonstrated anthocyanin biosynthesis in several plant species. In a prior study, the accumulation of anthocyanin and their associated gene expression were shown to be greatly increased in red orange fruit under low temperature storage condition [5]. In ripening grape berry skins, anthocyanin synthesis corresponds to a coordinated increased expression level of a number of anthocyanin biosynthetic genes [6]. It has also been revealed that the TTG1/bHLH/Myb transcriptional complex regulates the anthocyanin biosynthesis pathway in Arabidopsis seedlings [7]. A recent study combining transcriptome and metabolome analysis has shed light on pigment accumulation in peanut testa [8]. However, the underlying molecular mechanism between the peanut testa color and genes that regulate its related trait is still not clearly elucidated.

The biosynthesis of anthocyanin and its derivatives are catalyzed by a number of enzymes through the flavonoid pathway [9]. Initially, the phenylalanine amino acid is converted into 4-coumaryl-CoA with the help of phenylalanine ammonia-lyase (PAL), cinnamate 4-hydroxylase (C4H), and 4-coumaryl-CoA ligase (4CL). In the following step, 4-coumaryl-CoA and malonyl-CoA are sequentially catalyzed by different enzymes including chalcone synthase (CHS), chalcone isomerase (CHI), flavanone 3-hydroxylase (F3H), flavanone 3-hydroxylase (F3′H), and flavonoid 3′5′-hydroxylase (F3′5 ′H) to produce dihydrokaempferol. Then, colored anthocyanins pigments are synthesized from the colorless anthocyanins with the subsequent reactions of dihydroflavonol 4-reductase (DFR) and anthocyanidin synthase (ANS), and then converted into red, blue or purple glycosides by UDP-glucoside: flavonoid glucosyltransferase (UFGT) [10, 11]. Generally, genes-encoding key enzymes of anthocyanin pathway are highly expressed in fruits [12]. During the ripening process of a dark-colored fig fruit, the expression level of *FcANS1* gene was shown to be considerably increased [13]. Similarly, anthocyanin biosynthesis in grapes and strawberries relies on the upregulated expression of UFGT, which is identified as a key enzyme in anthocyanin pathway [14, 15]. Anthocyanins pathway genes are known to be activated at the transcription level by a ternary complex of MYB-bHLH-WD repeat (MBW complex) in many plant species [7, 16].

Peanut (*Arachis hypogaea* L.), is one of the important cash crops widely known for its high-quality edible oil and rich nutritional value around the world. Peanut testa is rich in amino acids, isoflavones, vitamins (B1, B3, B9, and E), anthocyanin, and procyanidins, which likely affect its economical and nutritional importance [17–19]. Peanut testa appear in a range of colors, the most frequent of which are pink and red. Other hues include purple, black, white, and multicolored testa. Different testa colored peanuts are considered to be linked with varied anthocyanin content and composition. For instance, the red testa is believed to be controlled by a single dominant gene present on chromosome 3, which causes the activation of anthocyanin biosynthesis [20]. In our previous findings, we also identified several up-regulated R2R3-MYB transcription factors associated with anthocyanin biosynthesis in black and pink testa peanut [19, 21]. In addition, our recent study also unleashed the control mechanism of red testa via a recessive gene, *AhRt2* located on chromosome 12 through BSA-seq and genetic mapping [22]. Previously, the high expression of genes such as *PAL*, *C4H*, *CHS*, and *CHI* as well as *AtMYB111* homologs may lead to the regulation of pink testa peanut [23]. The metabolome investigation of red and pink testa peanuts suggested that the content of petunidin and cyanidin in red testa was greater compared to the pink testa [8].

In recent years, high-throughput sequencing techniques have been used to investigate the development of pigmentation in a wide range of plant species by combining a number of omics-based methodologies. In fruit and flowers, metabolomic and transcriptome network have revealed a variety of secondary metabolites with altered content and their associated differentially expressed genes, expanding our understanding of how plants regulate their color [24, 25]. Using comparative transcriptome analysis, the significance of flavonoid biosynthesis and their regulatory genes have been investigated in black peanut testa [26]. Similarly, the gene expression profiling of important genes and transcription factors were studied during enhanced anthocyanin biosynthesis in red and yellow fruits of sweet cherries [27]. Another study used comparative transcriptome methods to compare the molecular mechanisms of changes in leaf and peel color found in red and green walnut [28]. Previously, the integrative analysis of multi-omics and miRNA profiling of pink and purple testa peanut uncovered important insights into the regulation of anthocyanin biosynthesis [29]. However, most of these studies were comparative analysis based on different peanut varieties with different testa colors. Besides testa color variation, there are many other trait differences among these varieties which can increase the number of DEGs. For this purpose, we used Bulk RNA-sequencing (BR-seq) analysis of red testa (ZH12) male parent and pink testa (Y9102) female parent with bulk F_4_ lines of red and pink testa peanuts. The findings of this study will broaden our knowledge regarding the genetic control mechanisms that regulate red and pink peanut testa color, and provide a valuable reference resource for studying gene-to-function relationship that governs pigmentation in peanut testa.

## Results

### Phenotypic variations and determination of anthocyanin content in red and pink testa peanut

**Fig. 1:**
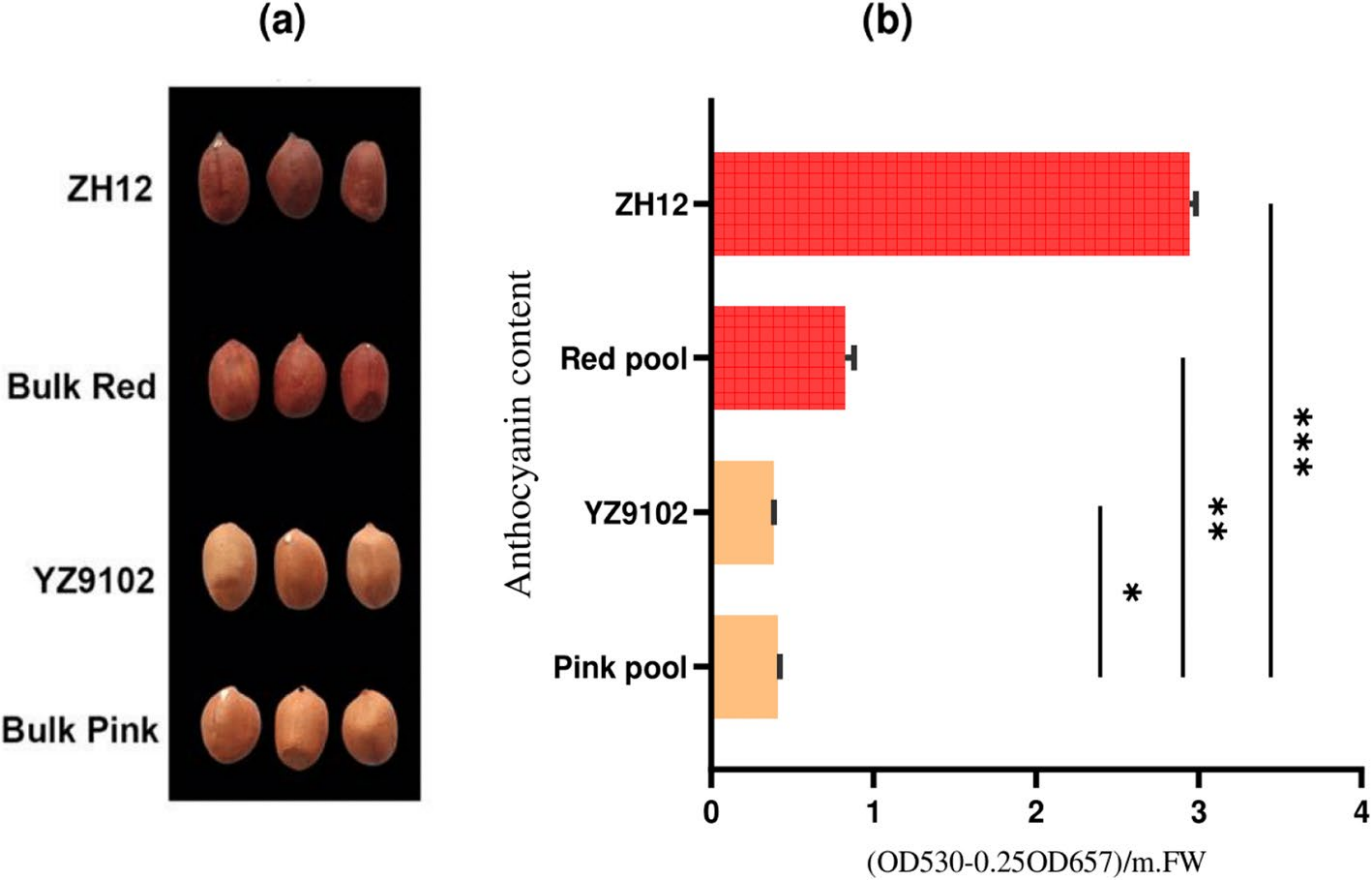
Phenotypic variations and determination of anthocyanin content (**a**) The peanut seeds demonstrating red testa parental line (ZH12) and pink testa parental lines (Y9102), with their counterpart bulk F_4_ lines. (**b**) relative anthocyanins content in parental lines and bulk F_4_ lines. The data were presented as SE (*n* = 3), and the asterisks * denotes *P* < 0.05, ** denotes *P* < 0.01 and *** denotes *P* < 0.001

The testa color of ZH12 is red and the testa of Y9012 is traditionally pink. The phenotypic investigation of red testa (ZH12) and pink testa (Y9012) cultivars with the bulk F_4_ lines of pink and red peanuts showed significant differences. The bulk F_4_ lines with red testa showed similar color with their counterpart male red parent (ZH12), whereas the bulk F_4_ lines with pink testa showed similar pink phenotype with their female parent (Y9012) counterpart (Fig. 1a). In addition, the measurement of total anthocyanin content of the fully matured seeds revealed approximately 2–7 times higher content in red testa peanut than the pink testa (Fig. 1b).

### Data filtration and reference genome mapping

A total of four cDNA libraries were constructed from ZH12, Y9102, bulk red and bulk pink peanuts, each one with three replicates, respectively. The BGISEQ-500 platform was used to sequence the cDNA libraries using the paired-end method. The raw reads were filtered by removing low quality, N-containing and adaptor-contaminated reads and approximately 21.04–22.47 million clean reads on average were acquired from the three replicates of each sample (Table 1). Further analysis showed that the average value of the clean reads Q20 (the proportion of filtered bases with a mass larger than 20 to the total number of bases) was 97.98%, while the average value of clean reads Q30 (the ratio of filtered bases with a mass value higher than 30 to the total number of bases) was 94.07%. The clean reads were mapped to the reference genome using HISAT software package and the results showed that the mapping ratio of each library showed 94.23–96.96% with a perfect average mapping ratio of 95.83%. The analysis of transcript readings using a random number generator revealed that the reads were scattered randomly across different regions of the transcript (Fig. S1). Similarly, it was found that the average proportion of each gene that was spanned by reads was more than 60% (Fig. S2). Hence, the RNA-seq data acquired from this platform might provide a precise reflection of high-quality data and could be applied to study expression analysis and follow-up analysis.

**Table 1:** Summary of the total read numbers obtained from BulkRed, BulkPink, Y9102 and ZH12 testa peanuts

Sample Name		Total Raw Reads (M)	Average Reads/sample (M)	Total Clean Reads (M)	Clean Reads (Q20, %)	Clean Reads (Q30, %)	Total Mapping (%)	Uniquely Mapping (%)
Pink	Pink-1	21.75	22.47	21.63	98.2	94.61	96.14	34.64
	Pink-2	21.75		21.63	97.8	93.56	96.33	35.17
	Pink-3	23.92		23.75	98	94.24	94.49	36.46
Red	Red-1	21.75	21.44	21.63	97.93	93.9	95.91	34.5
	Red-2	21.75		21.64	98.16	94.54	96.87	35.53
	Red-3	20.84		20.72	98.13	94.4	96.96	35.64
Y9102	Y9102–1	21.75	21.04	21.62	98.24	94.74	94.23	33.51
	Y9102–2	21.75		21.61	97.72	93.34	96.07	34.93
	Y9102–3	19.63		19.52	97.84	93.7	96.33	33.9
ZH12	ZH12–1	21.75	21.75	21.64	98.08	94.28	96.3	34.53
	ZH12–2	21.75		21.62	97.82	93.64	94.41	33.48
	ZH12–3	21.75		21.63	97.93	93.91	95.98	34.73

### Differential expressed genes (DEGs) identification between red and pink testa peanuts

**Fig. 2:**
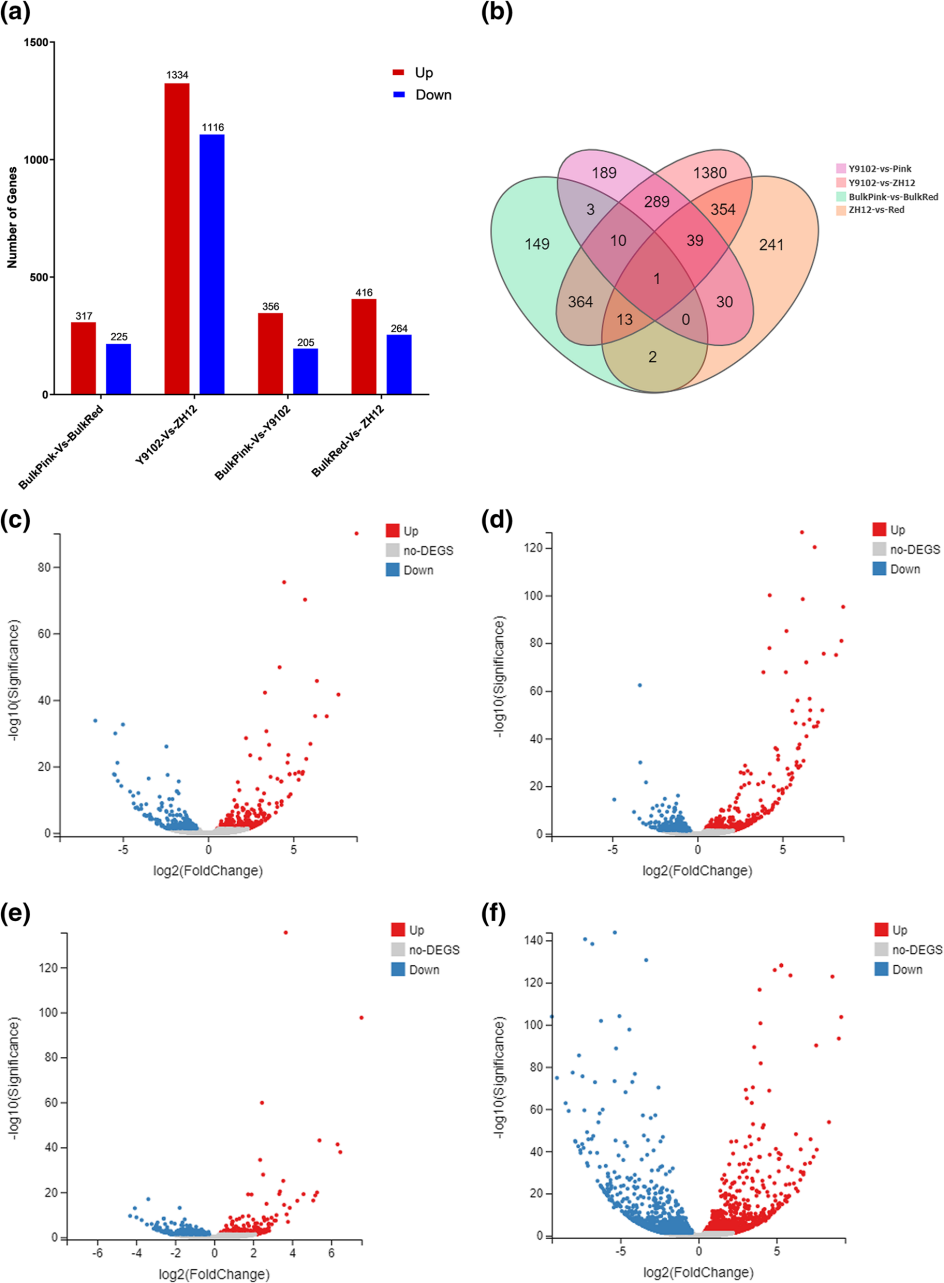
Significant changes of differentially expressed genes in in bulk red and bulk pink type peanuts compared to ZH12 (red parent) and Y9102 (pink parent) (**a**) Statistics of up-regulated and down-regulated differentially expressed genes (DEGs) in each experimental group (**b**) Venn diagram analysis of pink-vs-Y1902, red-vs-ZH12, ZH12-vs-Y9102 and bulk red-vs-bulk pink dataset. The volcano plot of the both up-regulated and down-regulated DEGs in (**c**) bulk pink-vs-Y1902 (**d**) bulk red-vs-ZH12 (**e**) bulk red -vs-bulk pink type peanuts (**f**) ZH12-vs-Y9102 type peanuts. The X-axis represents log_2_-transformed fold-difference values, and the Y-axis represents -log_10_-transformed significance values. Red dots represent up-regulated DEGs, blue dots represent down-regulated DEGs, and gray represent non-DEGs

In order to investigate the molecular mechanism governing the variation in red and pink testa color, we screened out DEGs using the following threshold log2 (FC) ≥ 1 and Q-value < 0.001 in bulk red, bulk pink, Y9102 and ZH12 testa peanuts. A total of 4233 DEGs were identified, among which 317 and 1334 in bulk red-vs-bulk pink were up-regulated and 225 and 1116 were down-regulated in Y9102-vs- ZH12 (Table S1 and S2). Similarly, 356 DEGs were up-regulated and 205 DEGs were down-regulated in bulk pink-vs-Y9102 group whereas 414 up-regulated and 264 down-regulated DEGs were detected in bulk red-vs-ZH12 group (Table S3 and S4). Noticeably, the number of up-regulated DEGs were slightly greater than the down-regulated genes between different experimental groups (Fig. 2a). Additionally, the analysis of Venn diagram indicated that the number of commonly expressed DEGs identified in different experimental groups. A total of 1089 DEGs in common were detected in bulk pink-vs-Y1902, bulk red-vs-ZH12, bulk red-vs-bulk pink and ZH12-vs-Y9102 groups, respectively (Fig. 2b). The illustration of Venn diagram depicts how these common or intersected DEGs could be relate to each other during the onset of testa color in peanut. It also elaborates to statistically compare different peanut groups (red and pink) within the same biological process by illustrating the common DEGs from each peanut group (overlap circles) and the individual DEGs that are exclusive to each peanut group (outer circles). Similarly, the volcano plot also enables the visual representation of biologically significant DEGs with large fold changes between each dataset following the negative binomial distribution principle (Fig. 2c-f).

### Functional enrichment analysis reveals crucial biological pathways during red and pink testa development

To gain deeper insights into the functions of discovered DEGs between different group of pink and red testa peanuts, the classification of gene ontology (GO) term(s) was carried out, which divided the identified DEGs into three functional terms: biological process, cell component, and molecular function (Table 2). The top 20 GO terms with the smallest Q-value or the selected GO Term (sorted by Q-value, up to 60) were sorted in all four group (Fig. S3). The GO enrichment results of differential genes in Y9102-vs-ZH12 group included hydrolase activity (GO:0016787), carbohydrate metabolic process (GO:0005975), cell periphery (GO: 0071944), and external encapsulating structure (GO:0016787) (Fig. S3a). In case of bulk pink-vs-bulk red group, the most significant enriched GO terms include regulation of gene expression (GO: 0010468), regulation of transcription, DNA-templated (GO: 0006355), secondary active transmembrane transporter activity (GO: 0015291), response to auxin (GO: 0009733), phototropism (GO: 0009638), and auxin-activated signaling pathway (GO: 0009734) (Fig. S3b). Similarly, the GO enriched term in bulk pink-vs-Y9102 classified into nucleobase-containing small molecule biosynthetic (GO: 0034404), signaling receptor binding (GO: 0005102), and glycerol ether metabolic process (GO: 0006662) (Fig. S3c). In the same way, the top GO enriched term in bulk red-vs-ZH12 include cell wall organization or biogenesis (GO: 0071554), external encapsulating structure organization (GO: 0045229), and external encapsulating structure (GO: 0030312) (Fig. S3d). Based on these observations, we speculated that DEGs enriched into different GO categories including response to auxin (GO: 0009733), phototropism (GO: 0009638), and auxin-activated signaling pathway could be crucial in understanding the underlying principles of peanut testa color regulation (Table S3).

**Table 2:**
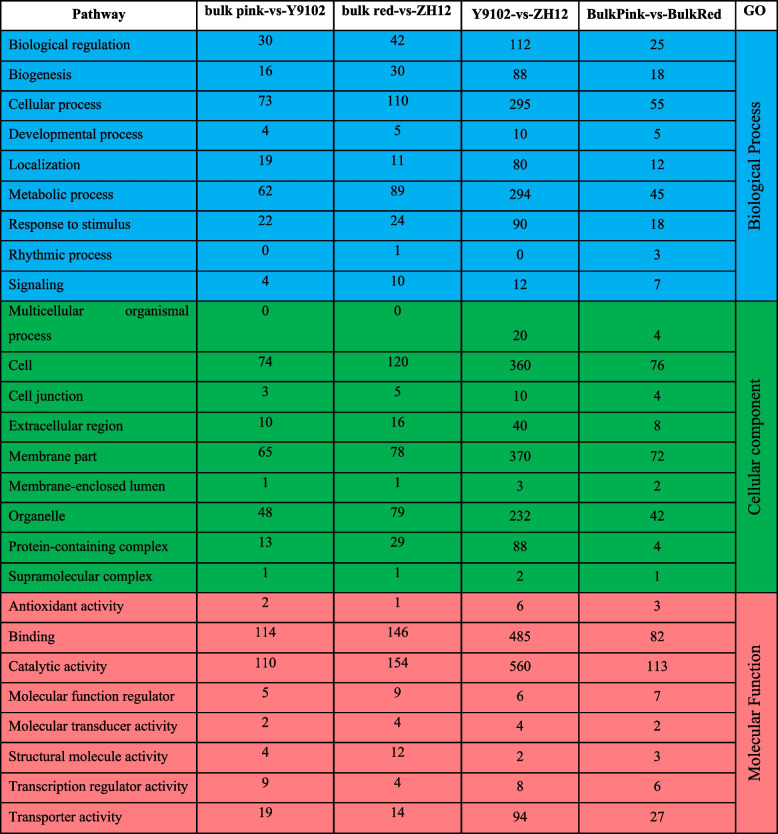
Significantly enriched GO terms and number of differentially expressed genes between all comparison group with pink and red testa peanuts

**Fig. 3:**
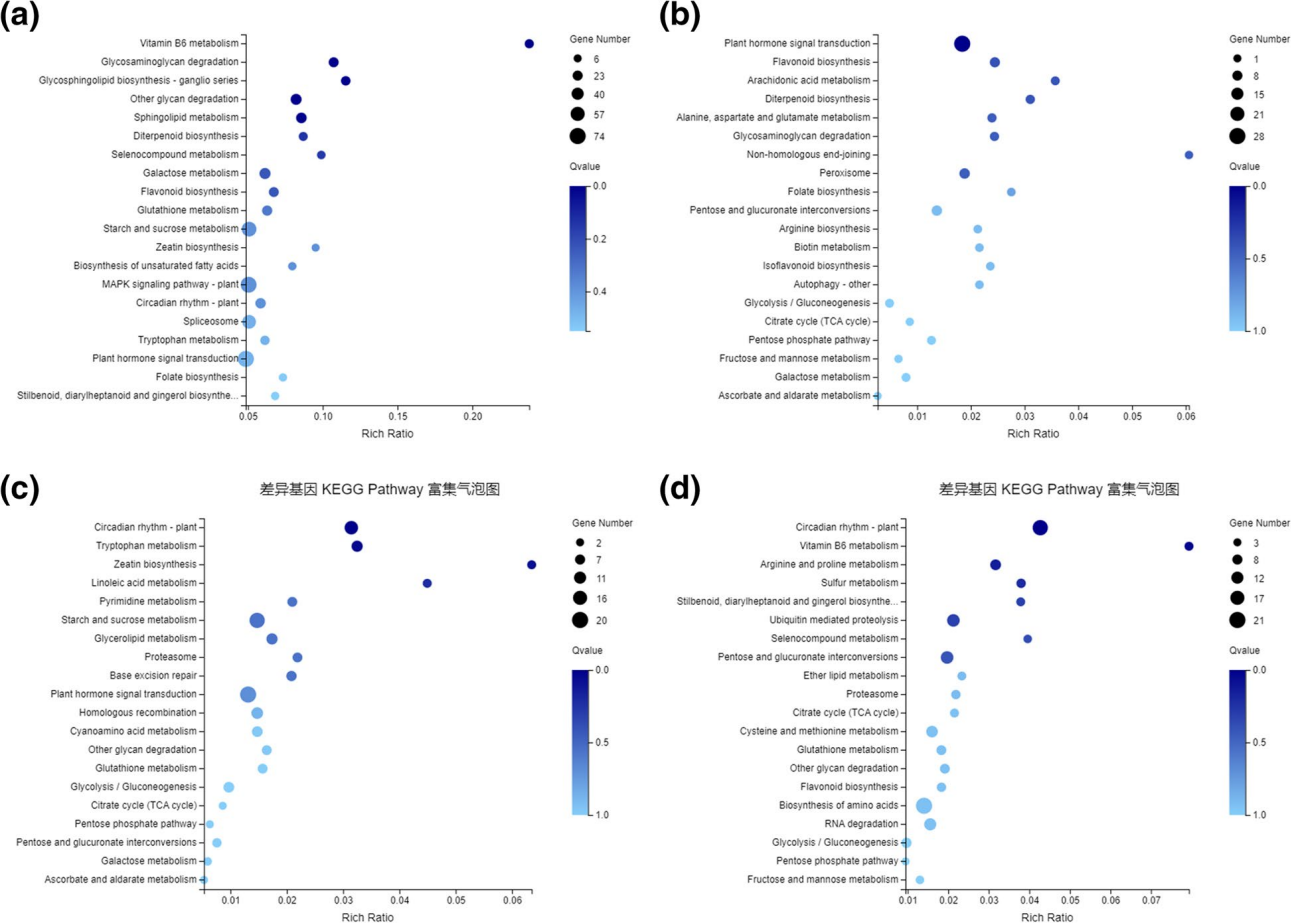
Elucidation of significantly enriched KEGG pathways involved during testa color in peanut. KEGG Pathway enrichment analysis based on the calculated *P*-value, and then the *P*-value was corrected for FDR, usually the function with Q-value <= 0.05 was regarded as a significant enrichment (**a**) Y9102-vs-ZH12 and (**b**) bulk pink-vs-bulk red testa peanuts (**c**) bulk pink-vs-Y1902 (**d**) bulk red-vs-ZH12. Source: www.kegg.jp/kegg/kegg1.html [30]

Furthermore, the KEGG pathway classification was carried out to obtain significantly enriched metabolic pathway between Y9102-vs-ZH12, bulk pink-vs-bulk red, bulk pink-vs-Y1902 and bulk red-vs-ZH12 testa peanuts. At first, the KEGG pathways sharing the identified DEGs between each group were categorized into five main branches including cellular processes, environmental information processing, genetic information processing, metabolism, and organic systems (Fig. S4). Further dissection of the enriched metabolic pathway in Y9102-vs-ZH12 indicated that a total of 1124 DEGs were enriched into approximately 129 functional pathways, whereas a total of 239 DEGs in bulk pink-vs-bulk red were significantly enriched into 102 KEGG pathways (Fig. 3a and b Table S4). Similarly, a total 255 DEGs into 95 KEGG pathways were detected in bulk pink-vs-Y9102 group whereas 303 DEGs were categorized into 102 KEGG pathways between bulk red-vs-ZH12 group (Fig. 3c and d, Table S5 and S6). Further analysis demonstrated that the functional pathways related to flavonoid biosynthesis (ko00941), isoflavonoid biosynthesis (ko00943), phenylpropanoid biosynthesis (ko00940), biosynthesis of other secondary metabolites (ko01110), plant hormone signal transduction (ko04075), diterpenoid biosynthesis (ko00904), carotenoid biosynthesis (ko00906) could be associated with the molecular regulation of testa color and these pathways were all significantly enriched in bulk pink-vs-bulk red compared to Y9102-vs-ZH12 group.

### DEGs involved in phenylpropanoid and flavonoid/anthocyanin biosynthetic pathways

**Fig. 4:**
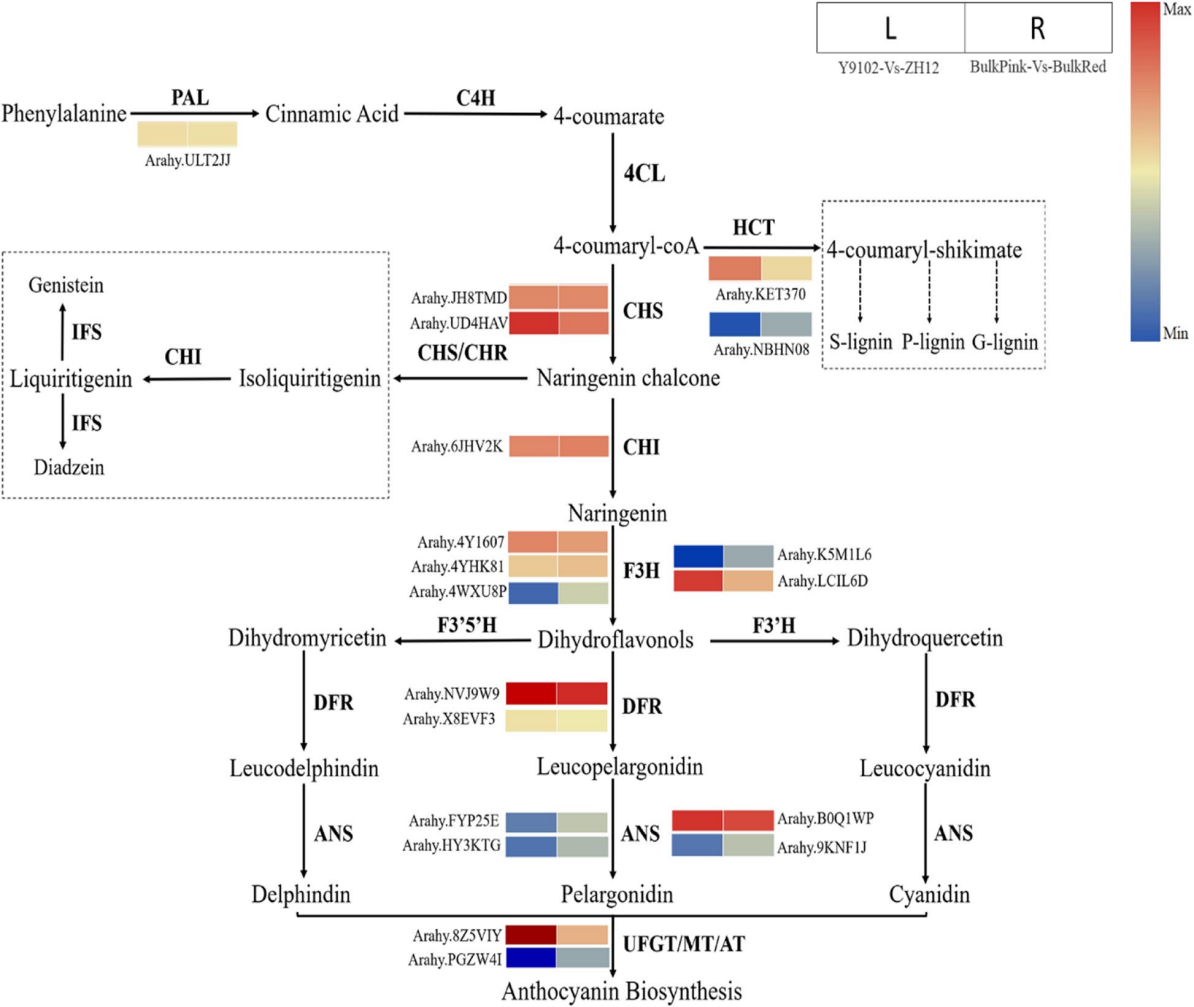
Reprogrammed expression of genes involved in the phenylpropanoid and flavonoid/anthocyanin biosynthetic pathways regulating red and pink testa development in peanut. The level of transcript abundance in the heatmaps was scored using log_2_-transformed fold-change values for each experimental group. PAL, phenylalanine ammonia lyase; C4H, cinnamate 4-hydroxylase; 4CL, 4-coumarate: CoA ligase; CHS, chalcone synthase; CHI, chalcone isomerase; CHR, chalcone reductase; F3H, flavanone 3-hydroxylase; IFS, 2-hydroxyisoflavanone synthase; F3’H, flavonoid 3′-hydroxylase: flavonoid 3′5′-hydroxylase; DFR, dihydroflavonol 4-reductase; ANS, anthocyanidin synthase; UFGT, UDP glucose-flavonoid 3-O-glcosyl-transferase; MT, methyltransferase

A total of 25 DEGs encoding core structural genes of the phenylpropanoid and flavonoid/anthocyanin biosynthetic pathway were identified between bulk pink-vs-bulk red testa group based on the KEGG pathway assignment (Fig. 4; Table S7). In total, the bulk pink-vs-bulk red testa group demonstrated 15 up-regulated and 10 down-regulated genes, whereas 16 up-regulated and 9 down-regulated genes were identified in Y9102-vs-ZH12 group. Mostly genes encoding *PAL*, *CHS*, *CHI*, *F3H*, *DFR* and *ANS* were preferentially expressed between the two testa peanuts. Interestingly, the expression level of one *PAL* encoding gene (*Arahy.X8EVF3*), two *CHS* encoding genes (*Arahy.JH8TMD* and *Arahy.UD4HAV*) and one *CHI* encoding gene (*Arahy.6JHV2K*) showed a similar up-regulation pattern of expression in both bulk pink-vs-bulk red testa and Y9102-vs-ZH12 testa group.

In addition, five *F3H* encoding genes, which catalyze the conversion of naringenin to different precursor molecules of dihydroflavonol derivatives in anthocyanin biosynthetic pathway were identified as differentially expressed genes. Two F3H genes (*Arahy.4WXU8P* and *Arahy.K5M1L6*) showed down-regulation in Y9102-vs-ZH12 testa and bulk pink-vs-bulk red testa groups, although their expression level was differently regulated by 3–4-fold change. Noticeably, the expression level of another *F3H* gene (*Arahy.LCIL6D*) showed 3-fold up-regulation in Y9102-vs-ZH12 testa groups compared to bulk pink-vs-bulk red testa group. The remaining two *F3H* genes (*Arahy.4Y1607* and *Arahy.4YHK81*) showed up-regulation in both testa groups with no significant difference in their expression level (Fig. 4, Table S7). *DFR* plays an important role in anthocyanin biosynthesis which mainly catalyzes different substrates to produce leucopelargonidin, leucocyanidin and leucodelphinidin. Our results identified two *DFR* encoding genes of which, one gene (*Arahy.NVJ9W9*) showed up-regulation in both testa groups, whereas one gene (*Arahy.X8EVF3*) was significantly down-regulated in the bulk pink-vs-bulk red testa compared to Y9102vsZH12 testa group. Similarly, four ANS encoding genes were identified between the two testa peanuts of which, two genes (*Arahy.B0Q1WP* and *Arahy.33FH8C*) demonstrated up-regulation while two genes (*Arahy.9KNF1J* and *Arahy.FYP25E*) showed down regulation in bulk pink-vs-bulk red testa and Y9102-vs-ZH12 testa peanut, respectively.

### DEGs involved in isoflavonoid biosynthetic pathway

**Fig. 5:**
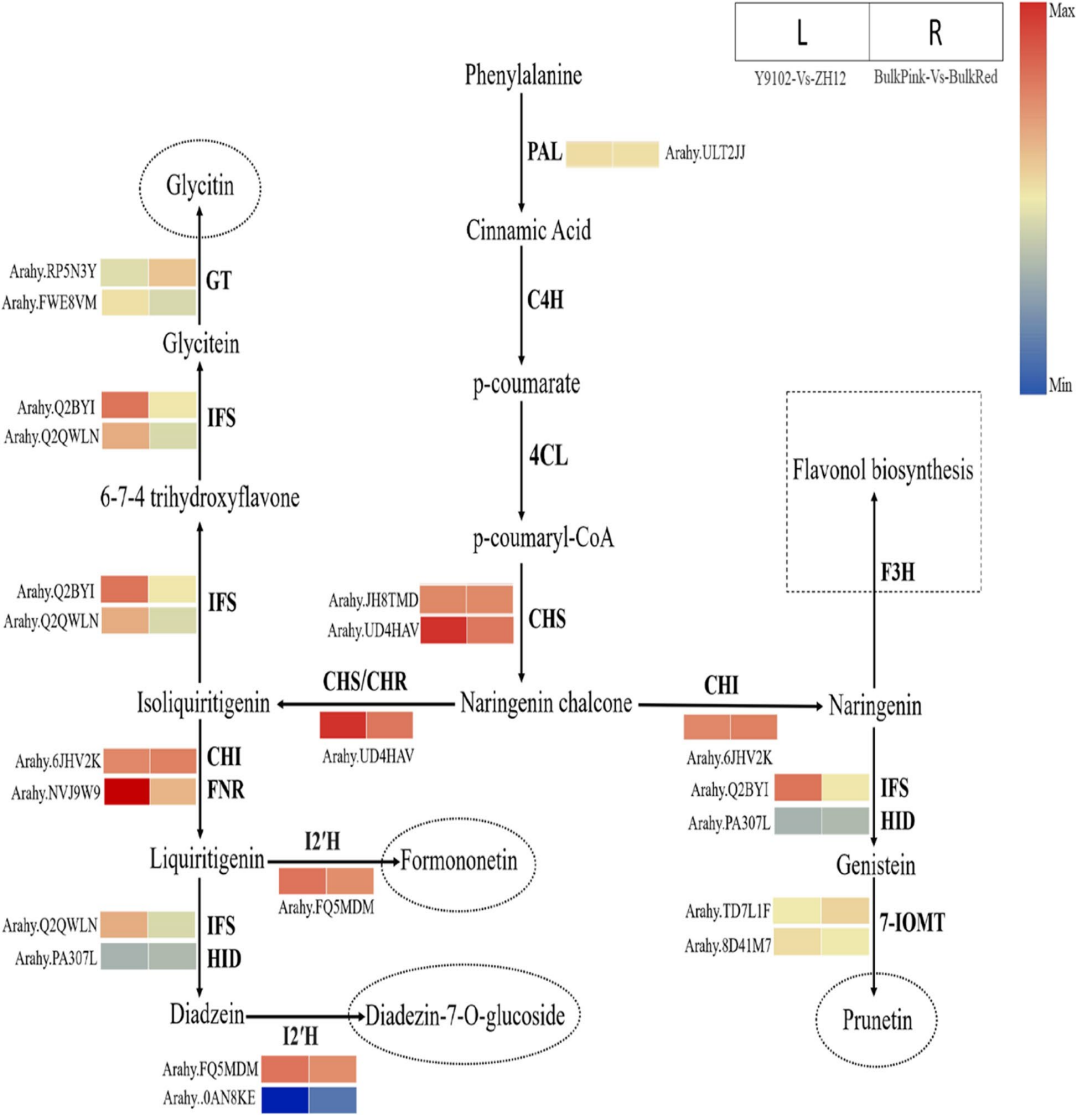
Reprogrammed expression of genes involved in the isoflavonoid biosynthetic pathway regulating red and pink testa development in peanut. The level of transcript abundance in the heatmaps was scored using log_2_-transformed fold-change values for each experimental group

During isoflavonoid biosynthetic pathway, chalcone synthase (CHS) co-acts with chalcone reductase (CHR) to produce isoliquiritigenin chalcone. Then, chalcone isomerase (CHS) or chalcone reductase (CHR) catalyze the conversion of isoliquiritigenin into liquiritigenin, thereby channeling the metabolic flux into isoflavone biosynthesis with the help of isoflavonone synthase (IFS) and 2-hydroxyisoflavanone dehydratase (HID). In this study, two genes encoding *IFS* were identified with differential expression in bulk pink-vs-bulk red testa and Y9102-vs-ZH12 testa peanuts. One gene encoding IFS (*Arahy*.*Q2QWLN*) showed up-regulation in Y9102-vs-ZH12, whereas the expression of *Arahy*.*Q2QWLN* was down-regulated in bulk pink-vs-bulk red. Another IFS encoding gene (*Arahy.Q2BYIT*) showed increased expression level up to (5-fold) in Y9102-vs-ZH12 compare to bulk pink-vs-bulk red peanuts (Fig. 5; Table S8). During isoflavonoid pathway, the HID enzyme generally catalyzes the conversion of liquiritigenin into and into daidzein and naringenin into genistein isoflavones. During our study, we also detected a HID-encoding gene (*Arahy.PA307L*), which showed down-regulation in both testa peanuts groups.

In the later steps of isoflavonoid pathway, isoflavone 2′-hydroxylase (I2’H) could convert liquiritigenin intermediate into formononetin and diadzein into diadzein-7-0 glucoside. Meanwhile, we found two genes encoding *I2’H*, of which one gene (*Arahy.FQ5MDM*) showed up-regulation, whereas another *I2’H* gene (*Arahy.0AN8KE*) showed down-regulation in Y9102-vs-ZH12 peanuts (Fig. 5; Table S8). Similarly, isoflavone-7-O-methyltransferase (7-IOMT) catalyze the conversion of genistein into prunetin during the down-stream regulation of isoflavone biosynthesis. Two genes encoding 7-IOMT were also identified with differential expression level between the two peanuts with different testa color (Fig. 5; Table S8).

### The crucial roles of transcription factors in the peanut testa color

**Fig. 6:**
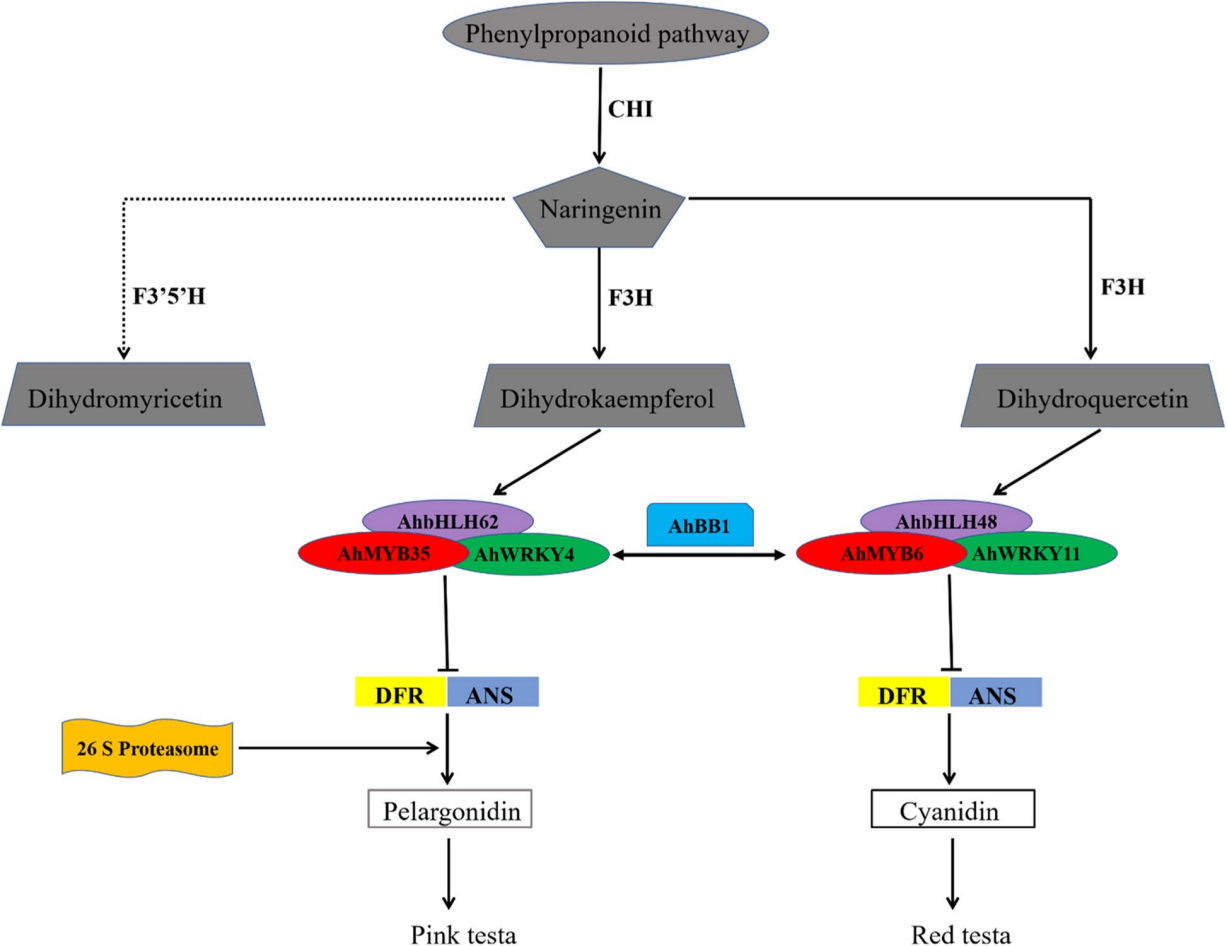
The proposed model of transcription factors-induced regulatory mechanism of peanut testa color development via anthocyanin biosynthetic pathway. The different colored oval shapes represent different types of transcription factors i.e. red; MYB, purple; bHLH and green; WRKY

In this study, a total of 13 *MYB* TF encoding genes, 9 *bHLH* TF differential expressed encoding genes were identified between Y9102-vs-ZH12 and bulk pink-vs-bulk red testa peanuts. Among them, 9 up-regulated and 4 down-regulated *MYB* TF encoding genes, 7 up-regulated and 2 down-regulated *bHLH* TF encoding genes), and 3 up-regulated and 4 down-regulated *WRKY* TF encoding genes were identified. Importantly, there is no *WD-40* TF encoding genes were detected, instead we detected 7 *WRKY* TF encoding genes in these two testa peanuts (Table S9). Further dissection of the four R2R3-MYBs (*Arahy.S8NMKG*, *Arahy.12J3VU*, *Arahy.WVAI17* and *Arahy.9UC92R*) showed that the expression changes between Y9102-vs-ZH12 and bulk pink-vs-bulk red peanuts. The expression level of *Arahy.S8NMKG* encoding a MYB6-like TF was significantly up-regulated in Y9102-vs-ZH12, whereas *Arahy.9UC92R* encoding *MYB35* like TF showed up to 11-fold down-regulation in ZH12 (Fig. 6; Table 3). Further comparison of their protein sequences revealed that *Arahy.S8NMKG* is highly homologous to *AtMYB5*, while *Arahy.9UC92R* shared homology with *TTG1* and *TT2* in Arabidopsis, which are known to be involved in anthocyanin biosynthesis and regulation of outer testa differentiation [31].

**Table 3:** Differentially expressed transcription factors between bulk red-vs-bulk pink and Y9102-vs-ZH12 peanuts

TF family	Gene name	Y9102-vs-ZH12	***P***-value	(bulk red-vs-bulk pink)	***P***-value	Nr Annotation
MYB	*Arahy.S8NMKG*	0.82	3.93E-05	−0.01	0.9758	XP_016205675.1|6.8e-231|transcription repressor MYB6 [Arachis ipaensis]
	*Arahy.12J3VU*	2.30	8.02E-12	0.60	0.2108	XP_025689501.1|1.4e-173|transcription repressor MYB6-like [*Arachis hypogaea*]
	*Arahy.WVAI17*	−3.70	1.68E-10	−0.39	0.5161	XP_025649931.1|2.1e-203|myb-related protein 306-like [*Arachis hypogaea*]
	*Arahy.9UC92R*	−3.14	4.30E-06	−0.29	0.6208	XP_016194299.1|1.8e-276|transcription factor MYB35-like [Arachis ipaensis]
WRKY	*Arahy.V3AFID*	−6.73	5.33E-51	−0.59	0.3347	XP_016194312.1|0.0e+ 00|probable WRKY transcription factor 4 [Arachis ipaensis]
	*Arahy.FP3Q5V*	0.54	0.0004	−0.13	0.6387	XP_025688450.1|7.6e-187|probable WRKY transcription factor 11 [*Arachis hypogaea*]
bHLH	*Arahy.GL41VM*	1.04	1.38E-06	0.22	0.4806	XP_016190307.1|2.1e-202|transcription factor bHLH61 [Arachis ipaensis]
	*Arahy.R1MRXV*	−2.78	1.27E-06	1.76	0.005	XP_025689410.1|5.5e-239|transcription factor bHLH62-like [*Arachis hypogaea*]
	*Arahy.26781 N*	2.31	6.90E-14	1.61	1.62E-06	XP_025634325.1|0.0e+ 00|basic helix-loop-helix protein A-like isoform X1 [*Arachis hypogaea*]
	*Arahy.SVL5FL*	2.19	4.42E-05	−1.79	5.44E-05	XP_025637498.1|3.2e-242|transcription factor bHLH48-like [*Arachis hypogaea*]

Furthermore, four *bHLHs* (*Arahy.GL41VM*, *Arahy.R1MRXV*, *Arahy.26781 N*, and *Arahy.SVL5FL*) also demonstrated preferential expression pattern between the two testa peanuts. The expression level of *Arahy.R1MRXV* encoding a *bHLH62* like TF was down-regulated in Y9102-vs-ZH12, whereas *Arahy.26781N* encoding a *bHLH48* like TF showed down-regulation in bulk pink-vs-bulk red (Fig. 6; Table 3). The alignment of their protein sequences showed close proximity with the Arabidopsis *AtTT8* encoding gene, which allows the regulation of cell-specific flavonoids accumulation [32]. In addition, two genes encoding WRKY transcription factors were also identified as differentially expressed genes between the two testa groups. One gene (*Arahy.FP3Q5V*) showed up-regulation in red peanuts and one gene (*Arahy.V3AFID*) showed down-regulation in red testa groups however. These findings provide important insights about the crucial role of transcription factors dependent regulation of peanut testa pigmentation. Together, these findings highlighted that the onset of testa color in peanut might involve the regulation of the core structural genes of anthocyanin biosynthetic pathway such as *DFR* and *ANS* by tipping off the MYB/bHLH/WRKY transcription factors.

### Transcriptomic data validated by quantitative real-time PCR assay

**Fig. 7:**
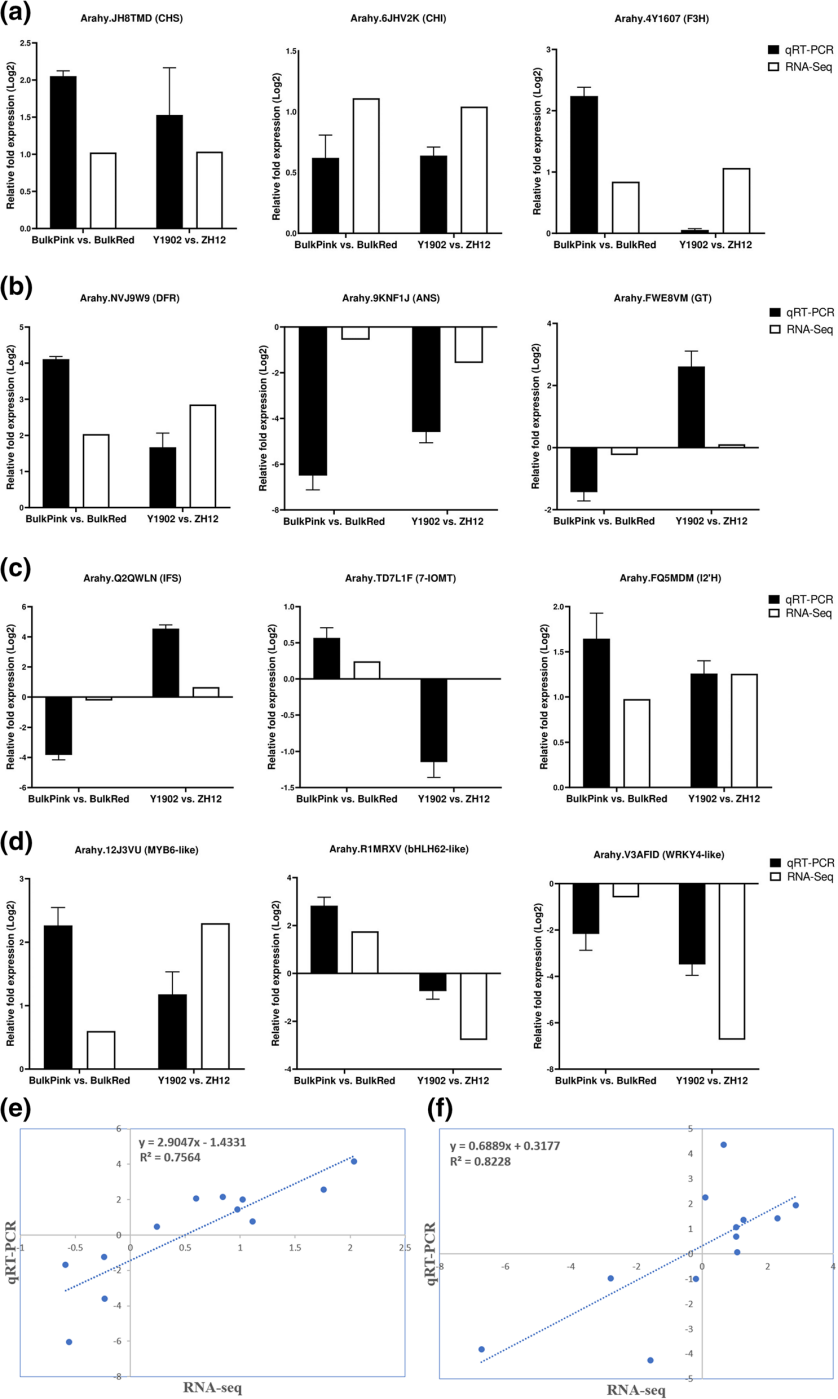
Validation of gene expression level of the key functional genes likely to be involved during the regulation of pink and red testa in peanut using qRT-PCR assay (**a**) DEGs selected from the up-stream regulatory anthocyanin pathway (**b**) DEGs selected from the down-stream regulatory anthocyanin pathway (**c**) DEGs selected from the iso-flavonoid regulatory pathway (**d**) DEGs encoding important transcription factors involved in anthocyanin pathway in peanut. Pearson’s correlation analysis of the RNA-seq and qRT-PCR in (**e**) bulk pink-vs-bulk red peanuts and (**f**) Y9102-vs-ZH12 peanuts. The black bars in the y-axis indicate the relative expression level of genes quantified by qRT-PCR, whereas the white bars indicate the expression results from the transcriptome data. The x-axis demonstrates the expression results obtained from bulk pink-vs-bulk red and Y9102-vs-ZH12 testa peanuts. The data were presented as means of three independent biological replicates, and error bars denote ± SE (*n* = 3)

To further confirm the integrity and reliability of RNA-seq data, a total of twelve genes including (9 phenylpropanoid and flavonoid/anthocyanin biosynthetic pathways genes, and 3 transcription factors) were selected and then investigated their transcript abundance in bulk pink-vs-bulk red and Y9102-vs-ZH12 testa peanuts using qRT-PCR analysis. As described in Fig. 7, the relative expression level of these functional genes showed a very similar pattern in both down and up-regulated gene expression with the transcriptome data of the two testa peanuts. For instance, the relative gene expression level of core structural genes of the flavonoid/anthocyanin biosynthetic genes such as *CHS, F3H, CHI, DFR and ANS* showed a consistent pattern of up and down-regulation in the two testa peanuts compared with the gene expression level of bulk RNA-seq results (Fig. 7). Similarly, the expression level of the functional genes involved in the isoflavonoid biosynthetic pathway including *IFS, 7-IOMT, I2’H* and *GT* also mirrored the expression pattern with the RNA-seq results of both testa peanuts. Importantly, three transcription factors (bHLH, MYB and WRKY) likely to be involved in the regulation of key anthocyanin biosynthetic genes, showed a consistent pattern of expression level with their transcriptomic results (Fig. 7). Based on these findings, we assume that quality, accuracy and reliability of the bulk RNA-seq data is sufficient for designing rational molecular studies in the future.

## Discussion

Anthocyanin is widely known as the water-soluble bioactive flavonoids abundantly found in deep-colored peanuts [17]. The accumulation level of anthocyanins was found to have a strong correlation with peanut pigmentation and nutrient quality. Several classes of flavonoids, such as anthocyanins and flavonols were identified in different peanut cultivars [17, 33]. Similarly, the testa of the two black peanut cultivars were analyzed for the identification of two anthocyanins classes and four types of flavonols [26]. The important role of flavonoid biosynthetic genes have been implicated during the regulation of black testa in peanut by utilizing comparative transcriptome profiling [26]. A previous study demonstrated that pink testa in peanut is regulated by the upregulation of genes encoding important structural genes of anthocyanin biosynthesis such as *PAL*, *C4H*, *CHS*, and *CHI* as well as transcription factor genes including *MYB*, *bHLH*, and *WRKY* [8]. Recent studies have shown that peanut seed contains several essential nutrients, and the color of the testa has a significant impact on the nutritional and economic value of peanuts [21, 34, 35]. Presently, red and pink testa containing peanuts are the most widely consumed types in China. Several efforts have been carried out to uncover the regulatory mechanism of testa colors in peanut [8, 21, 23, 36]. However, the existing knowledge is still limited to genome-wide and comparative transcriptomic and metabolome analysis. In this study, as an effort to elucidate a clear picture of the differential regulation of peanut testa color via flavonoid/anthocyanin biosynthesis, we established a bulk RNA-seq approach by comparing the bulk F_4_ generation of red and pink testa and their correspondent parents. Our findings provided in depth insights into the regulatory network that governs testa pigmentation and as a result, will aid in the development of improved peanut varieties through selective breeding.

### The orchestrated link between peanut testa color and anthocyanin metabolic flux

Plants produce anthocyanins, isoflavonoids, and flavonols, owing to a branch point of the phenylpropanoid system known as the flavonoid biosynthetic route. Among flavonoids, anthocyanins are the most important class which accumulate in different tissues (seeds, flowers, leaves and fruits) and contribute to their pigmentation, thus aiding seed and pollen dispersal [37]. In our study, we first analyzed the accumulation level of anthocyanin in red and pink testa peanuts along with their bulked F_4_ generation. The results suggested that the total anthocyanin content of the fully matured seeds of red testa peanut was higher than the pink testa peanut (Fig. 1B), indicating that the pigmentation of the peanut testa is strongly correlated with the accumulation level of anthocyanins. Previous studies confirmed that different plant species contained different level and types of anthocyanins resulting in multi-color hues. For instance, the integrated analysis of metabolomic and transcriptomic studies in green and purple fig cultivars showed significant differences in flavonoids metabolites in fruit peels [38]. It was further demonstrated that the cyanidin o-malonylhexoside was 3992-fold higher while different cyanidin glucosides were 100-fold higher in the mature purple peel, suggesting that anthocyanins are the key regulators of purple color in fig peels. Similarly, the transcriptome and metabolome analysis of jujube fruit peel during ripening periods also presented the underlying mechanism of red color formation. The reddening of jujube peel was shown to be strongly linked with the increase of malvidin and delphinidin content during ripening. It was also suggested that genes involved in the initial stages of the flavonoid biosynthetic pathway as well *UFGT* genes could also play important roles in anthocyanins accumulation when the fruit began the ripening process [39]. In addition, the acyl-modified type of anthocyanins are the most widespread class of anthocyanin found in Arabidopsis [40], whereas the only pigment detected in red leaves of cultivated lettuce is cyanidin 3-O-(malonyl)-glucoside [41]. A comparison of several asparagus varieties revealed that the major pigments in different varieties include, cyanidin, and peonidin type of anthocyanins [42]. Based on these informations, we further illustrate a deeper overview of the red and pink testa development in peanut with differently accumulated level of anthocyanins and thoroughly investigating the major alterations in gene-expression networks involved in anthocyanin biosynthetic pathway.

### The crucial role of the upstream flavonoid pathway during testa color development

The biosynthetic pathway of anthocyanins is generally regulated by a multi-enzyme complex in plants [9]. At first, the phenylpropanoid pathway is activated through the conversion of phenylalanine amino acid into 4-coumaryl-CoA by PAL, C4H and 4CL enzymes. Then, 4-coumaryl-CoA and malonyl-CoA are sequentially converted to naringenin and dihydrokaempferol through the action of CHS, CHI, F3H, F3′H, and flavonoid F3′5 ′H enzymes. At this stage, when naringin is converted into dihydrokaempferol, the anthocyanin biosynthesis pathway is activated. The colored anthocyanin pigments are then synthesized from their colorless precursor molecules using DFR, ANS, and UFGT enzymes into red, blue or purple glycosides [10, 11]. Noticeably, our results demonstrated the large-scale transcription expression changes of major flavonoid biosynthetic pathway genes in red and pink testa peanuts, supporting the existing paradigm of flavonoid/anthocyanin biosynthesis. Recently, studies have shown that upstream regulatory genes of the anthocyanin and flavonoid biosynthesis in blueberries and pears were found to be upregulated throughout the primary stages of fruit development [43, 44]. *CHS* has been discovered as key regulator of the red petal color in crabapple cultivars [45], whereas its expression was significantly inhibited in the colorless (white) petals in mustard crop, and the transcription of many other genes associated with anthocyanin biosynthesis pathway was comparable to that detected in the colored petals [11]. In peaches, the increased anthocyanin content of peaches was found to be associated with higher *CHS* expression [46]. In our study, we identified two *CHS* (*Arahy.JH8TMD* and *Arahy.UD4HAV*) with up-regulated expression in red testa peanuts, implying that the fundamental transcriptional regulation of the up-stream regulatory genes could occur far earlier than the development of the testa phenotype.

### Multi-channel regulation of core structural genes of anthocyanin pathway during peanut testa color formation

Transcriptional activation or inhibition of the *F3H* as well as the competitive expression of *FLS* and *DFR* genes particularly crucial in determining the production of anthocyanin component during anthocyanin pathway. Several studies have demonstrated that the synthesis of anthocyanins could be directed by the up-regulation of *F3′H* genes in *Rosa hybrida* and *Vitis vinifera* [47, 48]. The DFR derived from various plants have been shown to exhibit distinct substrate preferences for dihydrmyricetin, dihydroquercetin, and dihydrokaempferol [49, 50]. A previous study suggested that petunia flowers turn red when DFR enzyme catalyze the synthesis of pelargonin [51]. Recently, the positive role of DFR enzyme has been shown during the effective production of leucoanthocyanidin biosynthesis in safflower [52]. Pelargonin is accumulated in the petals of transgenic plants when maize *DFR* gene is introduced into white petunias, resulting in red petals [53]. Similarly, by overexpressing a number of *DFR* genes, the anthocyanin content in the flower tissues of tobacco was increased, which corresponded to a rise in red pigment [54]. Similar to other studies, our results also demonstrated that two F3H genes (*Arahy.4WXU8P* and* Arahy.K5M1L6*) were down-regulated in both pink testa and red testa peanuts, however, their expression level was differentially regulated by 3–4-fold change. Noticeably, the expression level of another *F3H* gene (*Arahy.LCIL6D*) showed 3-fold up-regulation in pink testa peanut (Fig. 4; Table S7). We found that one *DFR* (*Arahy.NVJ9W9*) showed up-regulation in both red testa groups. Furthermore, we found one *ANS* gene (*Arahy.33FH8C*) with up-regulated expression in red testa. While, one *ANS* gene (*Arahy.BOQ1WP*) showed up-regulation in both testa peanuts. These findings implied that the deepening of peanut testa color not only depends on the transcriptional activation of the key genes encoding *F3H, DFR*, and *ANS*, but also linked with the inhibition of *FLS* expression and bypassing flavonol biosynthesis.

### Regulation of peanut testa color via perturbation of lignin and isoflavone pathway

In plants, the biosynthesis of isoflavones and lignin both share a same upstream pathway with the common pathway of flavonoid. Hence, the regulatory pathway of anthocyanin is also depending on the inhibition of lignin and isoflavone synthesis. During the lignin biosynthetic pathway, a key enzyme Hydroxycinnamoyl: CoA transferase (HCT) competes with 4CL and catalyze the conversion of 4-coumaryl-CoA into 4-coumaryl-shikimate and then bifurcates the production of S-P-G type of lignin through the action of downstream regulatory enzymes [55]. The suppression of HCT gene in transgenic alfalfa showed a decreased content of lignin, resulting a clear shift in the composition of lignin [56]. In this study, we identified two *HCT* genes, of which one gene (*Arahy.NBHN08*) showed reduced expression level in ZH12 compared with Y9102. Isoflavanones, on the other hand, are produced by isoflavone synthase (IFS) and 2-hydroxyisoflavanone dehydratase (HID) enzyme using naringenin and/or liquiritigenin and then undergoes several tailoring steps such as glycosylation, methylation, and hydroxylation [57]. In a previous work, the agrobacterium rhizogenes transformation method was shown to confirm isoflavones accumulation in soybeans through the enhanced expression of *CHS* and *IFS* genes [58]. We also detected the reduced expression and/or down-regulation of *IFS*, *HID*, *7-IOMT* and *I2’H* in both testa peanuts, suggesting an obvious switch from isoflavone biosynthetic pathway towards anthocyanin biosynthesis.

### MYB–bHLH with a co-option of WRKY transcription factors regulatory model in peanut testa color

In combination with known functional genes, flavonoid/anthocyanin biosynthesis is transcriptionally regulated by MBW complex [16]. Similarly, anthocyanin and flavonoid biosynthetic genes are regulated by MYB and bHLH transcription factors in numerous plant species [59–61]. One of the most important roles of MYB TFs is to regulate anthocyanin pathway genes, owing to pigmentation in fruits and flowers [45, 62]. Similar findings were found for the pear *PpMYB10* and *PpMYB114*, which are responsible for regulating anthocyanin biosynthesis [63]. In Arabidopsis, MYB TFs have been reported, of which *PAP1* and *PAP2* shared important role as the common transcription factors to regulate the transcription of main anthocyanin structural genes [7]. Recently, the expression of the two R2R3-MYB genes, namely* AhMYB1* and *AhMYB2* was significantly increased in black peanuts [26]. Previously, we reported the identification of candidate gene (*AhTc1*) encoding a R2R3-MYB TF regulates the testa color in purple peanuts [19]. In this study, the expression level of MYB6-like TF (*Arahy.S8NMKG*) demonstrated high homology with the Arabidopsis (*AtMYB5*) and was significantly up-regulated in Y9102-vs-ZH12 testa peanut.

Basic helix-loop-helix (bHLH) TFs, are known to govern control the expression of genes such as DFR, ANS, and ANR in the seedlings and pods of Arabidopsis [64]. The bHLH TF also participates in the assembly of MBW complex, which regulates both anthocyanin and procyanidin synthesis in Arabidopsis, Petunia hybrid, *Antirrhinum majus* and *Zea mays* [7, 31, 65–67]. In this study, the expression level of *Arahy.R1MRXV* encoding a bHLH62 like TF was significantly down-regulated in Y9102-vs-ZH12, whereas *Arahy.26781 N* encoding a bHLH48 like TF showed up-regulation in both bulk pink-vs-bulk red and Y9102-vs-ZH12 testa peanuts. However, no such genes that code for anthocyanin-related WD-40 containing transcription factors were identified in both peanut testa groups. The findings were found consistent with previous findings, who provides the underlying network in black peanut testa color development [26]. Furthermore, and one gene (*Arahy.V3AFID*) showed down-regulation in red testa peanut ZH12. Previous, researches have shown that a WRKY TF (*TTG2*) plays an important role in the development of trichome and testa formation in Arabidopsis [68]. Recent research has shown that the WRKY-based regulation mechanism is present in Petunia but absent in Arabidopsis [69]. Nevertheless, in contrast to *tt2* and *tt8* mutants, the color morphology of *ttg2* mutants is confined to the testa and no deficit of anthocyanins was detected in the plant body of *ttg2* mutants. Hence, we proposed that *WRKY* TF alone could not sufficiently and directly induce anthocyanin biosynthesis in peanut testa, however, the co-expression of *MYB* and *bHLH* TFs could stimulate the transcription of *WRKY* encoding genes. In conclusion, the co-option of *WRKY*-induced MYB-bHLH regulatory complex could provide a fascinating insight into the regulation of peanut testa color development via anthocyanin biosynthesis.

## Conclusions

The present study describes the identification of key functional genes that are most likely involved during the regulation of testa color in red and pink-types peanuts using a combination of BGISEQ-500 platform and bulk RNA-seq technology. Our findings revealed that the expression of *PAL*, *CHS*, *CHI*, *F3H*, *DFR,* and *ANS* encoding genes could be the key players governing the deepening of peanut testa color. In addition, the down-regulation of *HCT*, *IFS*, *HID*, *7-IOMT* and *I2’H* genes suggested an alternative route for anthocyanin accumulation through perturbation of lignin and isoflavone pathway. In addition, the role of MYB, bHLH, and WRKY transcription factors also provides an intriguing transcriptional activation module in deciphering the underlying regulatory mechanism of peanut testa color via anthocyanin biosynthesis.

## Materials and methods

### Plant materials and phenotypic analysis

Peanut cultivars including red testa Zhonghua12 (ZH12), and pink testa Yuanza9102 (Y9102) were kindly provided by oil crop research institute of Chinese Academy of Agricultural Sciences. The two cultivars Zhonghua12 (ZH12), and Yuanza9102 (Y9102) along with F_2:4_ lines of population YZZH12 (Y9102 x ZH12) were grown in the experimental field located at Shandong Academy of Agricultural Sciences, Jinan, China under the controlled agricultural conditions. The population of YZZH12 was constructed using single seed descent which has been described in our previous study [22]. The phenotypic homozygous F_4_ lines were selected for mixing RNA pools as the method describe in our previous study [70]. The seeds of ZH12 and Y9102 were further selected, and allowed to grow until maturity. Similarly, the F_4_ bulk red and bulk pink lines were obtained, respectively. The testa was carefully excised from the seeds of both pink and red peanuts at fully mature stage and then analyzed for further investigation.

### Anthocyanin quantification

Three sets of more than eight peanut seeds per sample were used to quantify the anthocyanin content of each testa peanut, following the previously reported protocol with only small alterations [19]. The anthocyanin content of peanut seeds was measured at a development stage of 50 days after pegging. Briefly, 50 mg of frozen seeds were ground to an extremely fine powder in liquid nitrogen. Then, 700 μl of acidic methanol (99:1) was added to the homogenized testa, and the extraction was performed at 4 °C. Following an overnight incubation time, the homogenates were subjected to centrifugation for 10 minutes at 12000 rpm at room temperature. The filtrates (roughly 600 μl) was separated and thoroughly mixed with 1 mL of trichloromethane and 400 μl of distilled water. The mixture was then centrifuged for 10 minutes at 4 °C and 12,000 rpm. With the help of a spectrophotometer (U-3000, HITACHI, Japan), the absorbance of the supernatant was measured at 530 and 657 nm. The relative anthocyanin content was calculated using the absorbance eq. [A530–0.25 × A657)/FW] unit/mg Fresh Weight. and then adjusted by weight of the sample. Three biological replicates were chosen from each plant material (ZH12, Y9102, bulk red and bulk pink lines) for RNA-seq analysis.

### Library construction and RNA sequencing

The total RNA content was isolated from the testa with Trizol Reagent kit (TaKaRa, Inc., Dalian, China) following the manufacturer’s protocol. The purity and amount of the RNA from each sample was determined by Agilent 2100 and NanoDrop. The total RNA was treated with mRNA enrichment method using polyA tail connected magnetic beads with Oligo (dT). The DNA/RNA hybrid strand was selectively digested by RNaseH, and then the DNA probe was digested by DNaseI, and the desired RNA was obtained after purification. The mRNA was treated with fragmentation buffer to cleave into short fragments. Reverse transcription with random N6 primers was carried out, and then double-stranded DNA was synthesized. The double-stranded DNA was linearized and phosphorylate the 5′ end, and produce a sticky end with an “A” protruding from the 3′ end, and finally connected to a bubble-shaped linker with a protruding “T” at the 3′ end. The ligated product was then amplified by PCR using specific primers. The PCR products obtained from the previous step were thermally denatured into single-stranded DNA, and then a single-stranded circular DNA library was obtained by circularizing the single-stranded DNA with the help of a bridge primer. After that, sequencing was carried out using DNBSEQ platform.

### Data analysis and reference genome alignment

Raw reads were thoroughly filtered using the in-house filtering software SOAPnuke (v1.5.2) (https://github.com/BGI-flexlab/SOAPnuke) to obtain the clean reads. The removal reads of reads containing adapters (adapter contamination), reads with unknown base N content greater than 10%, and low-quality reads (the bases with a quality value below 15 accounting for more than 50% of the total bases in the read) was conducted. Finally, the filtered clean reads were assembled in a FASTQ file. After obtaining the clean reads, we used HISAT (Hierarchical Indexing for Spliced Alignment of Transcripts) [71] to align the clean reads to the reference genome sequence of *Arachis hypogaea* cv. Tifrunner (https://www.peanutbase.org/data/public/Arachis_hypogaea/).

### Identification of differentially expressed genes (DEGs)

High-quality clean reads were aligned to the reference gene sequences using Bowtie2 software package (http://bowtie-bio.sourceforge.net/Bowtie2/index.shtml) [72]. Then, the expression level of genes and transcripts were calculated from fragments per kb per million reads (FPKM) values using RSEM software [73]. The R package of DEseq2 was used to perform DEG detection based on the negative binomial distribution principle following the instructions described by [74], and the *P*-values were adjusted according to normal distribution. The (DEGs) were scored based on the thresholds log2 (FC) ≥ 1 and Q-value < 0.001.

### Gene ontology (GO) and KEGG enrichment analysis of DEGs

To investigate the functional annotation, the DEGs identified in different groups of red and pink testa peanuts were mapped to the corresponding Gene Ontology (GO) terms and Kyoto Encyclopedia of Genes and Genomes (KEGG) pathways analysis. Then, the classification of DEGs was performed with GO functional terms and biological pathways using the phyper function in R software for enrichment analysis. The *p*-value was calculated according to the following method:

$$\textrm{P}=1-\sum_{i-0}^{m-1}\frac{\left(\begin{array}{c}M\\ {}i\end{array}\right)\left(\begin{array}{c}N-M\\ {}n-i\end{array}\right)}{\left(\begin{array}{c}N\\ {}n\end{array}\right)}$$

The *p*-values were then FDR corrected and typically features with Qvalue <= 0.05 were considered significantly enriched.

### Transcription factors and plant disease resistance encoding DEGs

For plant transcription factors, the ORF of Unigene was identified by getorf, and then used hmmsearch (http://hmmer.org/) to align the ORF to the transcription factor protein domain (data from TF). The Unigene’s ability was identified according to the transcription factor family characteristics described in PlantTFDB. Similarly, the DIAMOND software (https://github.com/bbuchfink/diamond) was used to align the genes to the plant disease resistance gene database PRGdb for annotation, and the annotation results were further screened according to conditions such as query coverage and identity, in order to obtain possible disease resistance genes.

### Validation of RNA-seq data using qRT-PCR

The expression results of twelve selected unigenes were chosen for confirmation with qRT-PCR assay. The total RNA content was extracted from individual samples and the first strand cDNA templates were prepared using the PrimeScript II reverse transcriptase system (TaKaRa). Primers for each unigene were designed using Primer3 software. The experiment was performed using the qRT PCR system ABI7500 Real Time System (Applied Biosystems) using SYBR Green I (Roche). Each reaction was carried out in a total of 20 μL volume following the reaction conditions: 94 °C 10 min; 94 °C 15 s, 60 °C 10 s and 72 °C 25 s for 40 cycles. Three independent biological replicates were used for each sample and the actin gene was kept as an internal reference gene. The relative expression of genes was calculated by using the 2 − ^△△Ct^ method.

## Supplementary Information

**Additional file 1: Fig. S1.** The random reads distribution of bulk pink, bulk red, Y9102 and ZH12 samples. **Fig. S2.** Reads coverage of bulk pink, bulk red, Y9102 and ZH12 samples. **Fig. S3.** GO enrichment analysis. The enrichment bubble chart shows the enrichment degree of GO Term from three dimensions. By default, the top 20 GO Term with the smallest Qvalue or the selected GO Term (sorted by Q-value, up to 60) are plotted. The figure below shows the GO enrichment results of differential genes in (a) Y9102-Vs-ZH12 and (b) bulk pink-vs-bulk red peanuts. **Fig. S4.** KEGG Pathway Classification. The KEGG metabolic pathway is divided into 7 branches: Cellular Processes, Environmental Information Processing, Genetic Information Processing, Metabolism, Organic Systems. (a) Y9102-Vs-ZH12 and (b) bulk pink-vs-bulk red peanuts. **Fig. S4.** Multiple sequence alignment between Arahy.S8NMKG and AtMYB5. **Fig. S5.** Multiple sequence alignment between Arahy.9UC92R and TTG1 / TT2.

**Additional file 2: Table S1.** Statistics of up-regulated and down-regulated differentially expressed genes (DEGs) in each comparison group.

**Additional file 3: Table S2.** Expression level of DEGS in all comparison groups with red and pink testa peanuts.

**Additional file 4: Table S3.** Significantly enriched GO terms between different comparison groups with pink and red testa peanuts.

**Additional file 5: Table S4.** KEGG Pathway enrichment analysis between different comparison groups with pink and red testa peanuts.

**Additional file 6: Table S5.** DEGS distribution into different KEGG pathways between bulk pink-vs-bulk red testa peanuts.

**Additional file 7: Table S6.** DEGS distribution into different KEGG pathways between Y9102-vs-ZH12 testa peanuts.

**Additional file 8: Table S7.** KEGG pathway enrichment of DEGs involved in phenylpropanoid and flavonoid/anthocyanin biosynthetic pathways.

**Additional file 9: Table S8.** KEGG pathway enrichment of DEGS involved in iso-flavonoid pathway.

**Additional file 10: Table S9.** Expression level of Transcription factors encoding genes in bulk pink-vs-bulk red and Y9102-vs-ZH12 testa peanuts.

## Data Availability

The datasets generated and/or analyzed during the current study are available in the Sequence Read Archive (SRA repository), under the Accession number PRJNA886491 [https://www.ncbi.nlm.nih.gov/sra/PRJNA886491] at NCBI.

## Abbreviations

DEGs: Differentially expressed genes
KEGG: Kyoto Encyclopedia of Genes and Genomes
GO: Gene ontology
FDR: The False Discovery Rate
qRT-PCR: Quantitative real-time PCR
TF: Transcription factor
μl: Microliter
mg: Milligram
rpm: Revolutions per minute
nm: Nanometer
