# Supplementary material for: Transcriptional networks orchestrating red and pink testa color in peanut
Source: BMC Plant Biol. 2023 Jan 19;23:44. doi: 10.1186/s12870-023-04041-0 (PMC9850581; doi:10.1186/s12870-023-04041-0)

**Fig. S1.** The random reads distribution of bulk pink, bulk red, Y9102 and ZH12 samples.


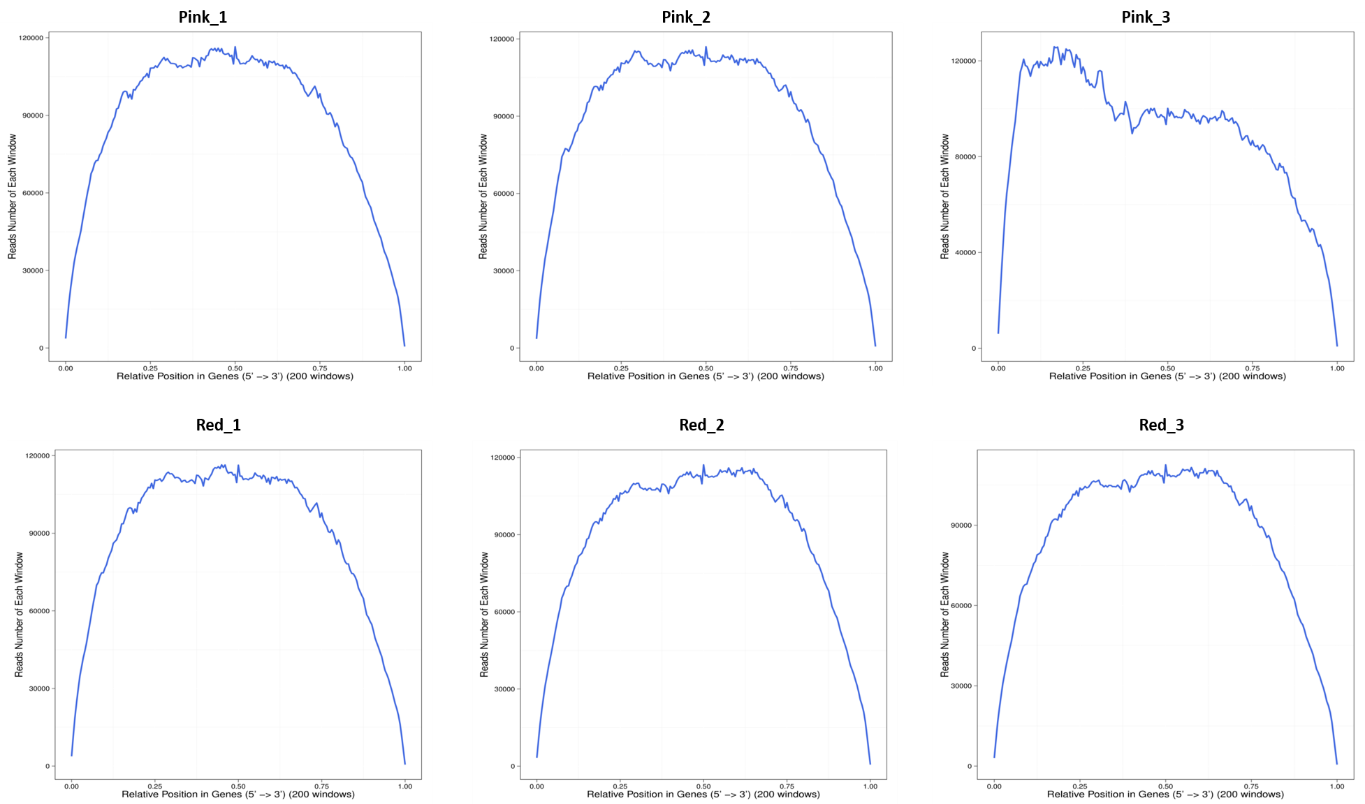


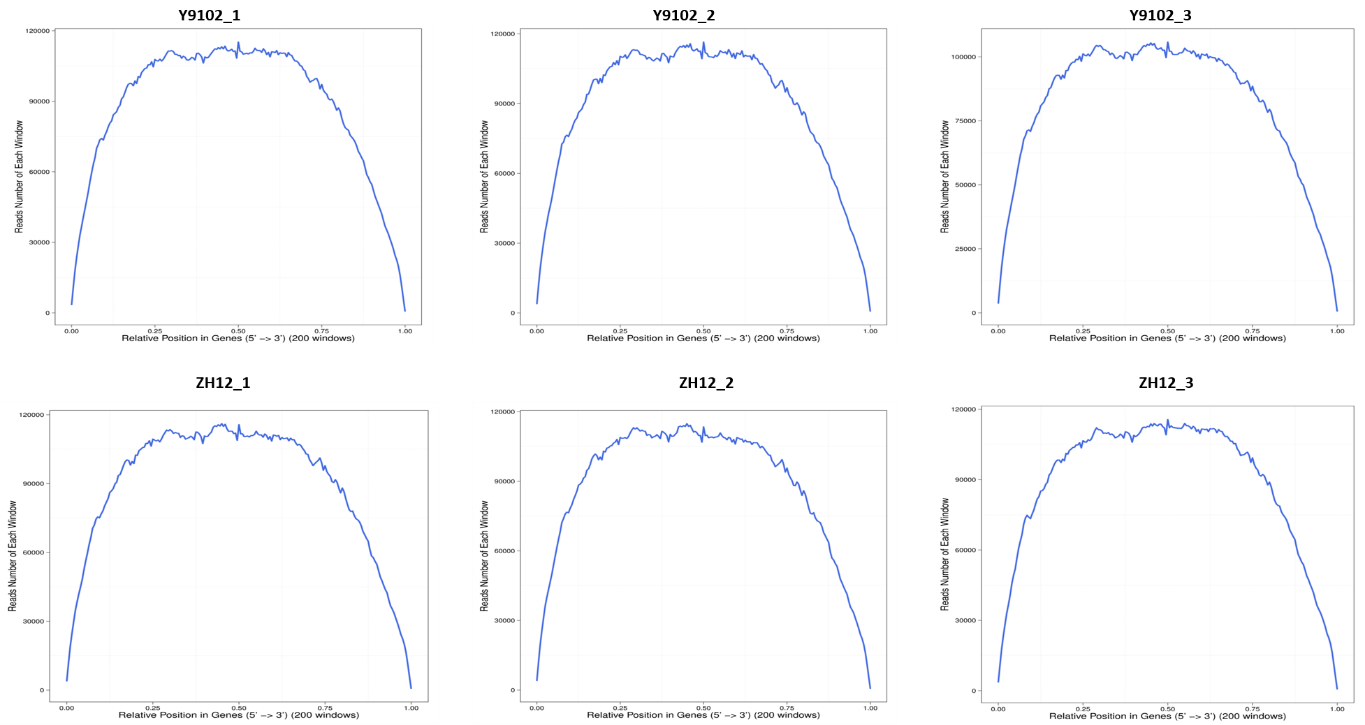


**Fig. S2.** Reads coverage of bulk pink, bulk red, Y9102 and ZH12 samples.


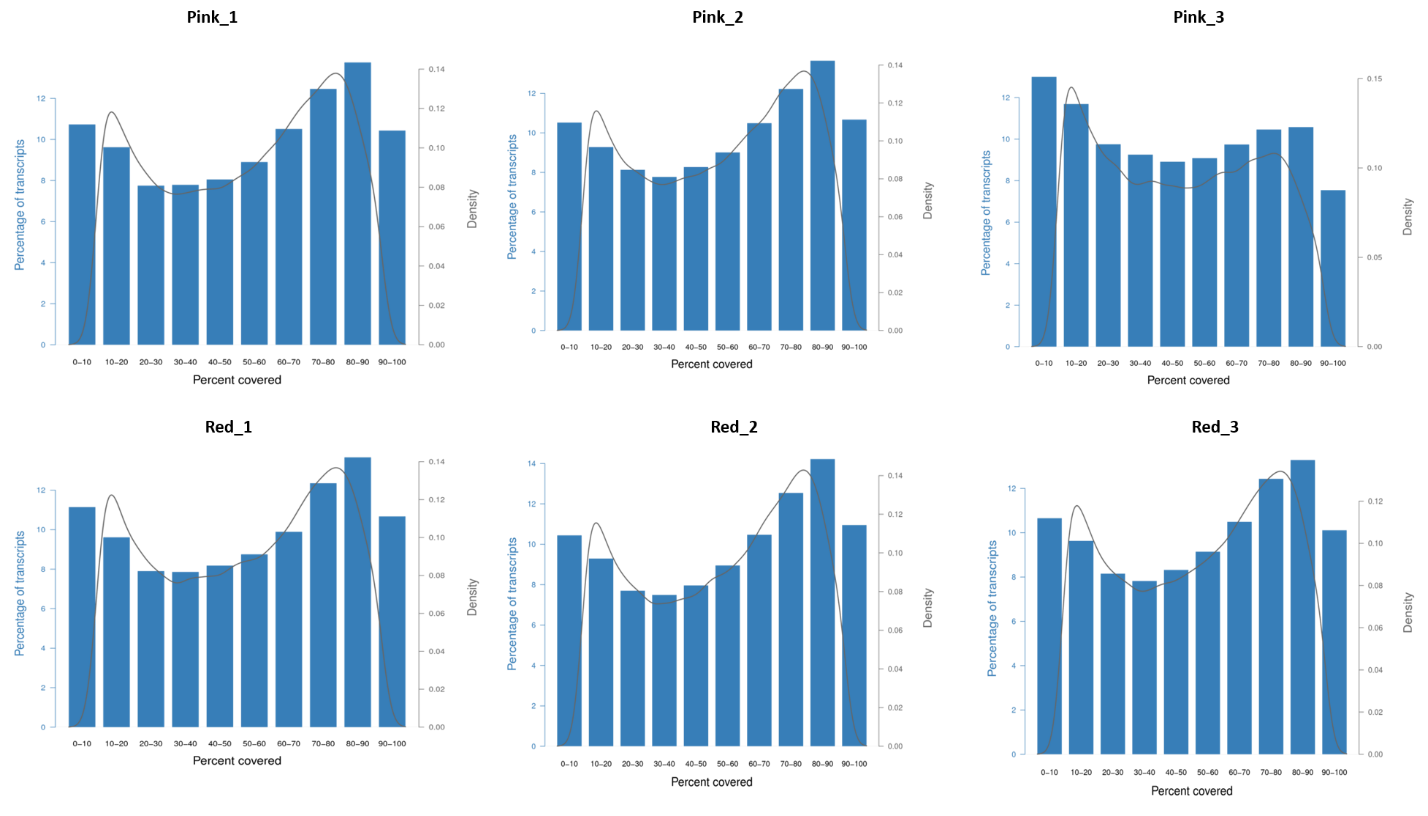


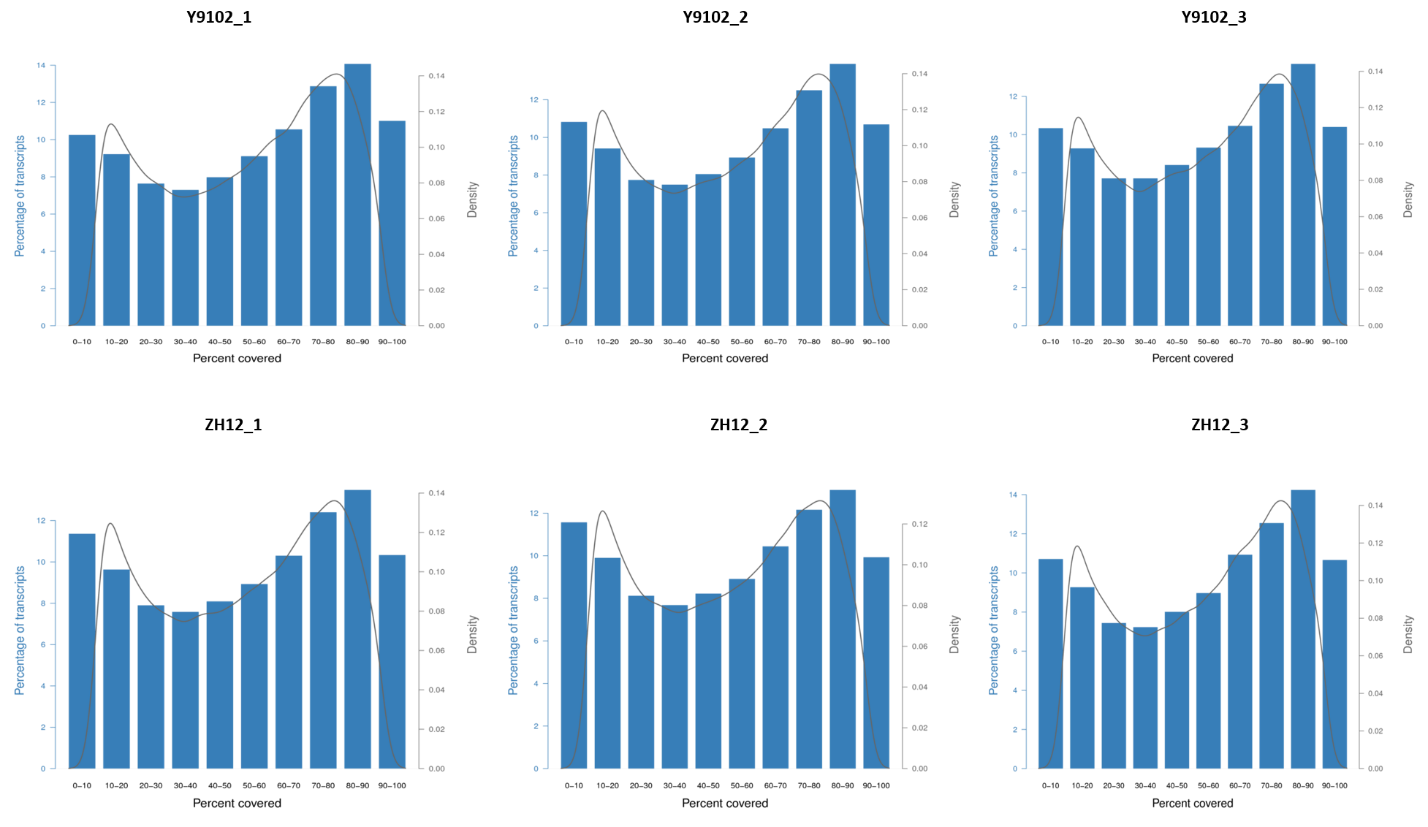


##### Fig. S3. GO enrichment analysis. The enrichment bubble chart shows the enrichment degree of GO Term from three dimensions. By default, the top 20 GO Term with the smallest Qvalue or the selected GO Term (sorted by Q-value, up to 60) are plotted. The figure below shows the GO enrichment results of differential genes in (a) Y9102-Vs-ZH12 and (b) bulk pink-vs-bulk red peanuts.

1. **Y9102-Vs-ZH12 testa peanuts**


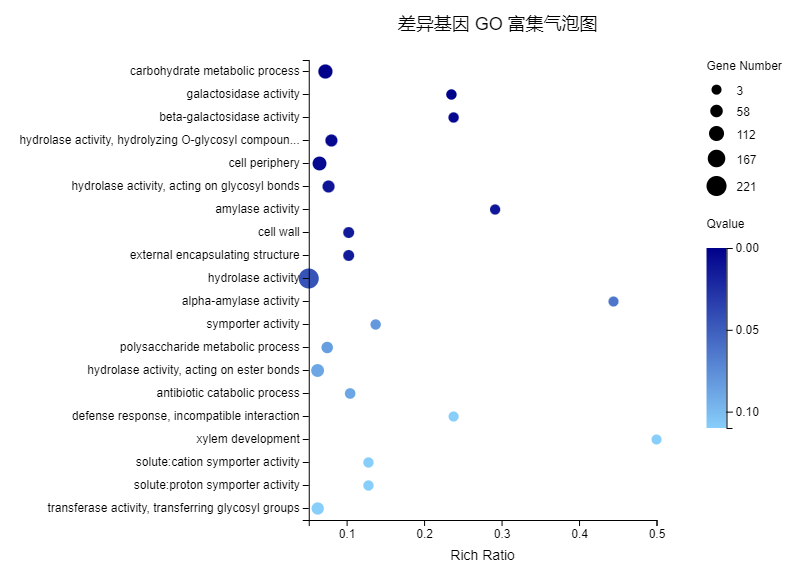


1. **BulkPink-Vs-BulkRed testa peanuts**


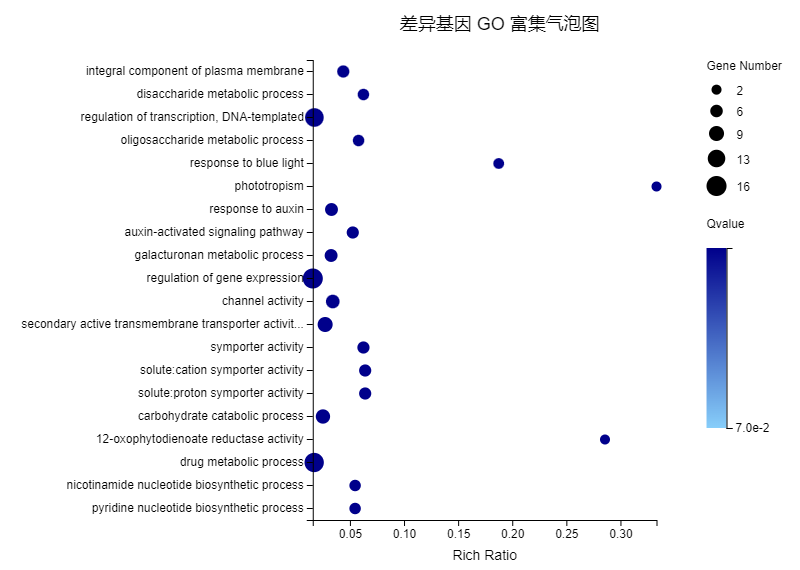


##### Fig. S4. KEGG Pathway Classification. The KEGG metabolic pathway is divided into 7 branches: Cellular Processes, Environmental Information Processing, Genetic Information Processing, Metabolism, Organic Systems. (a) Y9102-Vs-ZH12 and (b) bulk pink-vs-bulk red peanuts.

1. **Y9102-Vs-ZH12 testa peanuts**


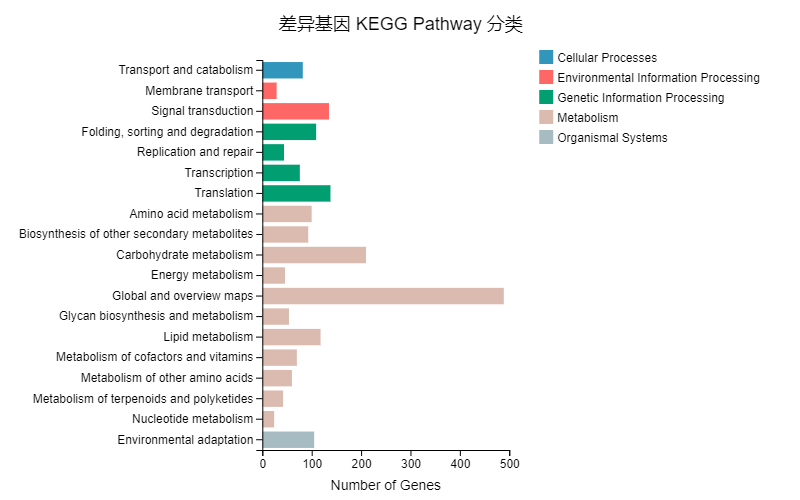


1. **BulkPink-Vs-BulkRed testa peanuts**


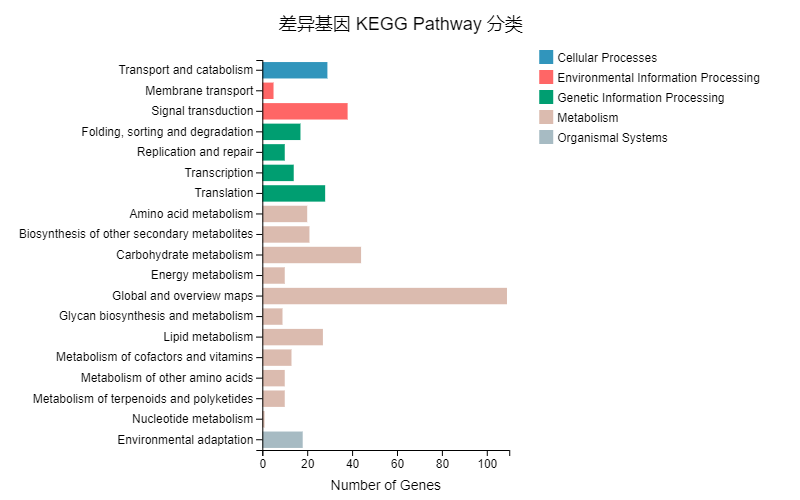


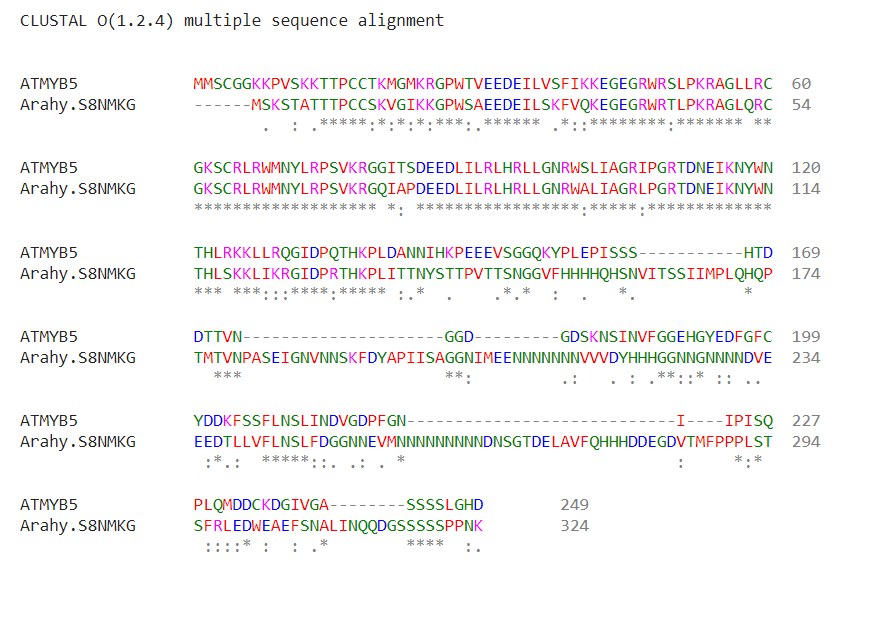
**Figure S4:**Multiple sequence alignment between Arahy.S8NMKG and AtMYB5.

**Figure S5:**Multiple sequence alignment between Arahy.9UC92R and TTG1 / TT2.


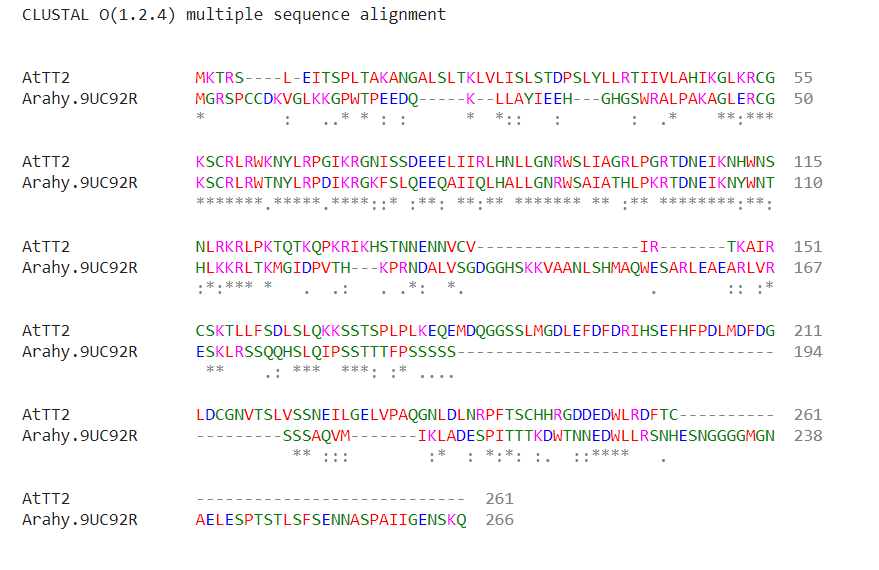

Supplement: Supplementary file 1 — Additional file 1: Fig. S1. The random reads distribution of bulk pink, bulk red, Y9102 and ZH12 samples. Fig. S2. Reads coverage of bulk pink, bulk red, Y9102 and ZH12 samples. Fig. S3. GO enrichment analysis. The enrichment bubble chart shows the enrichment degree of GO Term from three dimensions. By default, the top 20 GO Term with the smallest Qvalue or the selected GO Term (sorted by Q-value, up to 60) are plotted. The figure below shows the GO enrichment results of differential genes in (a) Y9102-Vs-ZH12 and (b) bulk pink-vs-bulk red peanuts. Fig. S4. KEGG Pathway Classification. The KEGG metabolic pathway is divided into 7 branches: Cellular Processes, Environmental Information Processing, Genetic Information Processing, Metabolism, Organic Systems. (a) Y9102-Vs-ZH12 and (b) bulk pink-vs-bulk red peanuts. Fig. S4. Multiple sequence alignment between Arahy.S8NMKG and AtMYB5. Fig. S5. Multiple sequence alignment between Arahy.9UC92R and TTG1 / TT2. [file 12870_2023_4041_MOESM1_ESM.docx]
